# An orbital strategy for regulating the Jahn–Teller effect

**DOI:** 10.1093/nsr/nwae255

**Published:** 2024-08-05

**Authors:** Tongtong Shang, Ang Gao, Dongdong Xiao, Qinghua Zhang, Xiaohui Rong, Zhexin Tang, Weiguang Lin, Ting Lin, Fanqi Meng, Xinyan Li, Yuren Wen, Xuefeng Wang, Dong Su, Zhen Chen, Yong-Sheng Hu, Hong Li, Qian Yu, Ze Zhang, Lijun Wu, Lin Gu, Jian-Min Zuo, Yimei Zhu, Liquan Chen, Ce-Wen Nan

**Affiliations:** State Key Laboratory of New Ceramics and Fine Processing, National Center for Electron Microscopy in Beijing, School of Materials Science and Engineering, Tsinghua University, Beijing 100084, China; Beijing National Laboratory for Condensed Matter Physics, Institute of Physics, Chinese Academy of Sciences, Beijing 100190, China; State Key Laboratory of New Ceramics and Fine Processing, National Center for Electron Microscopy in Beijing, School of Materials Science and Engineering, Tsinghua University, Beijing 100084, China; Beijing National Laboratory for Condensed Matter Physics, Institute of Physics, Chinese Academy of Sciences, Beijing 100190, China; Beijing National Laboratory for Condensed Matter Physics, Institute of Physics, Chinese Academy of Sciences, Beijing 100190, China; Songshan Lake Materials Laboratory, Dongguan 523808, China; Beijing National Laboratory for Condensed Matter Physics, Institute of Physics, Chinese Academy of Sciences, Beijing 100190, China; Beijing National Laboratory for Condensed Matter Physics, Institute of Physics, Chinese Academy of Sciences, Beijing 100190, China; Beijing National Laboratory for Condensed Matter Physics, Institute of Physics, Chinese Academy of Sciences, Beijing 100190, China; Beijing National Laboratory for Condensed Matter Physics, Institute of Physics, Chinese Academy of Sciences, Beijing 100190, China; Beijing National Laboratory for Condensed Matter Physics, Institute of Physics, Chinese Academy of Sciences, Beijing 100190, China; State Key Laboratory of New Ceramics and Fine Processing, National Center for Electron Microscopy in Beijing, School of Materials Science and Engineering, Tsinghua University, Beijing 100084, China; Beijing National Laboratory for Condensed Matter Physics, Institute of Physics, Chinese Academy of Sciences, Beijing 100190, China; School of Materials Science and Engineering, University of Science and Technology Beijing, Beijing 100083, China; Beijing National Laboratory for Condensed Matter Physics, Institute of Physics, Chinese Academy of Sciences, Beijing 100190, China; Beijing National Laboratory for Condensed Matter Physics, Institute of Physics, Chinese Academy of Sciences, Beijing 100190, China; State Key Laboratory of New Ceramics and Fine Processing, National Center for Electron Microscopy in Beijing, School of Materials Science and Engineering, Tsinghua University, Beijing 100084, China; Beijing National Laboratory for Condensed Matter Physics, Institute of Physics, Chinese Academy of Sciences, Beijing 100190, China; Beijing National Laboratory for Condensed Matter Physics, Institute of Physics, Chinese Academy of Sciences, Beijing 100190, China; Department of Materials Science and Engineering, Center of Electron Microscopy and State Key Laboratory of Silicon Materials, Zhejiang University, Hangzhou 310027, China; Department of Materials Science and Engineering, Center of Electron Microscopy and State Key Laboratory of Silicon Materials, Zhejiang University, Hangzhou 310027, China; Condensed Matter Physics and Materials Science Division, Brookhaven National Laboratory, New York, NY 11973, USA; State Key Laboratory of New Ceramics and Fine Processing, National Center for Electron Microscopy in Beijing, School of Materials Science and Engineering, Tsinghua University, Beijing 100084, China; Beijing National Laboratory for Condensed Matter Physics, Institute of Physics, Chinese Academy of Sciences, Beijing 100190, China; Department of Materials Science and Engineering, University of Illinois at Urbana Champaign, Urbana, IL 61801, USA; Condensed Matter Physics and Materials Science Division, Brookhaven National Laboratory, New York, NY 11973, USA; Beijing National Laboratory for Condensed Matter Physics, Institute of Physics, Chinese Academy of Sciences, Beijing 100190, China; State Key Laboratory of New Ceramics and Fine Processing, National Center for Electron Microscopy in Beijing, School of Materials Science and Engineering, Tsinghua University, Beijing 100084, China

**Keywords:** quantitative convergent-beam electron diffraction, Jahn–Teller effect, transition-metal oxide, local coordinate strategy, orbital degenerate

## Abstract

The Jahn–Teller effect (JTE) arising from lattice–electron coupling is a fascinating phenomenon that profoundly affects important physical properties in a number of transition-metal compounds. Controlling JT distortions and their corresponding electronic structures is highly desirable to tailor the functionalities of materials. Here, we propose a local coordinate strategy to regulate the JTE through quantifying occupancy in the ${{d}_{{{z}^2}}}$ and ${{d}_{{{x}^2} - {{y}^2}}}$ orbitals of Mn and scrutinizing the symmetries of the ligand oxygen atoms in MnO_6_ octahedra in LiMn_2_O_4_ and Li_0.5_Mn_2_O_4_. The effectiveness of such a strategy has been demonstrated by constructing P2-type NaLi*_x_*Mn_1__–_*_x_*O_2_ oxides with different Li/Mn ordering schemes. In addition, this strategy is also tenable for most *3d* transition-metal compounds in spinel and perovskite frameworks, indicating the universality of local coordinate strategy and the tunability of the lattice–orbital coupling in transition-metal oxides. This work demonstrates a useful strategy to regulate JT distortion and provides useful guidelines for future design of functional materials with specific physical properties.

## Teaser

This work provides a general orbital engineering strategy to regulate the Jahn-Teller distortion and will inspire continued efforts to design structures with different physical properties for functional materials.

## INTRODUCTION

The Jahn–Teller effect (JTE) describes the symmetry-lowering geometrical distortion of non-linear molecules with degenerate electronic ground states, which is most often encountered in the octahedral complexes of transition metals (TM), such as manganese (Ⅲ) and copper (II) complexes with electron unevenly occupied *e_g_* orbitals [1–4]. In considering strongly correlated functional oxide materials, the JTE usually leads to coupling between the lattice, charge, orbital and spin degrees of freedom [5,6] and gives rise to various phenomena [7–9]. For example, JT distortion not only plays a crucial role in stabilizing A-type antiferromagnetic ordering [2], but also has been invoked in explaining charge ordering in manganites [10]. The JTE of Cu^2+^ has also been confirmed to be an important component of high-temperature superconductivity in copper oxides [4,11,12]. As a result, great progress has been achieved in investigating JT distortion and its modulation by external fields, such as temperature [13], strain [14–16] and electric fields [17,18]. Previous experiments to identify the JTE mainly relied on X-ray diffraction (XRD) or neutron diffraction, which focus on the crystal symmetry-lowering and phase transition induced by JTE. These above techniques often need complementary experiments to prove the change in electronic structures induced by JTE, such as electrical transport measurement, magnetic susceptibility measurement or electron energy loss spectroscopy [13,19,20]. As the JTE describes the lifting of orbital degeneracy, giving rise to electron redistribution among *d* orbitals, the most direct method to probe JTE is to quantify the *d*-orbital populations of TM ions. The shape of valence electron density in real space can intuitively show the electron accumulation and depletion around bonding regions and deformation around TM in line with *d*-orbital energy splitting. Real-space valence electron density measurement can explore the JTE and provide clues to its regulation. Experimental determination of electron density usually relies on high-precision XRD [21,22], electron diffraction [23,24] and scanning tunneling microscopy [25,26]. Among these techniques, quantitative convergent-beam electron diffraction (QCBED) can accurately measure low-order structure factors that are sensitive to valence electrons. With complementary high-order structure factors obtained from density functional theory (DFT) calculations or XRD, the electron density can be reconstructed through multipole refinement of both low-order and high-order structure factors (as seen in Equation S1). Also, the *d*-orbital electron populations can be determined from multipole parameters, thereby realizing the detection of JTE at the orbital level.

As we know, Mn-based cathode materials of lithium-ion batteries (LIBs) are very attractive for application in large-scale energy storage and electric vehicles because of their advantages of low cost and environmental friendliness [27,28]. However, the practical use of Mn-based cathodes has been hindered by the cooperative JTE associated with high-spin Mn^3+^, which causes a phase transition from cubic to tetragonal in spinel structures, or from rhombohedral to monoclinic in layered structures [29] during cycling, aggravating the structural irreversibility due to the large lattice changes. Besides, as one of the products of the disproportionation reaction of Mn^3+^, Mn^2+^ can easily dissolve into the electrolyte, leading to structural degradation and rapid capacity fading. Therefore, effective strategies to suppress the JTE of Mn ions would be helpful to enhance the electrochemical performance of Mn-based oxide cathodes. In this work, we select spinel LiMn_2_O_4_ as a prototype to show that real-space measurement of orbital electron population can give us new insights to guide the regulation of the JT distortion. For this purpose, we accurately measured the *3d*-orbital electron populations for Mn ions in MnO_6_ octahedra of Li*_x_*Mn_2_O_4_ (*x *= 0.5 and 1) and found equally occupied electrons for the three *e_g_* and two *t_2__g_* orbitals, respectively, indicating the degeneracy of the *d* orbital and hence no JTE in both samples. Through a careful survey of the electron density of ligand oxygen atoms around Mn in Li*_x_*Mn_2_O_4_, we found that the equivalence of oxygen atoms is highly related to the degeneracy of the *d* orbital. Based on the above findings, we proposed a local coordinate strategy to regulate the JT distortion from the point view of orbitals, by controlling the local symmetry of the ligand oxygen atoms in the MnO_6_ octahedron. Moreover, we demonstrated the effectiveness and universality of this local strategy in the modulation of JT distortion in TM oxides.

## ORBITAL DEGENERACY OF Mn IN SPINEL Li*_x_*Mn_2_O_4_

**Figure 1.**
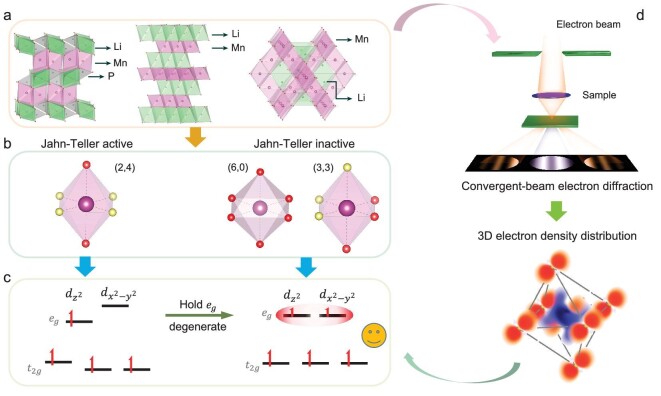
Orbital strategy to control the Jahn–Teller effects (JTE). (a) Three common structure models (olivine, layered, spinel) of cathode materials for rechargeable Li-ion battery. (b) MnO_6_ octahedra with different symmetries. Ligands with the same color are identical and vice versa. The left octahedron is JT active, while the right two octahedra are JT inactive. (c) The orbital strategy to maintain the degeneracy of *e_g_* levels to suppress the JTE. (d) By combining convergent-beam electron diffraction and density functional theory (DFT) calculations, *d*-orbital occupancy can be quantified, and therefore the JTE can be detected.

To describe the symmetry of the coordinated oxygen atoms of the MnO_6_ octahedron in typical Mn-based cathode materials as shown in the left panel of Fig. 1a–c, we introduced the (*n*, 6 − *n*) concept, in where *n* indicates the number of oxygen atoms having the same charge state in the MnO_6_ octahedra. In a regular octahedron, the six coordinated oxygen atoms are equivalent, which can be denoted as a (6, 0) configuration (Fig. 1b). When Li ions are extracted from or inserted into the Mn-based oxide framework, the six coordinated oxygen atoms may no longer be equivalent and hence can be divided into *n* O1 atoms and (6 − *n*) O2 atoms, in which *n* can be 5, 4, 3. Among these configurations, the coordinate axes of the MnO_6_ octahedron are identical for the (6, 0) and (3, 3) symmetries (Fig. S1), enabling the electrons to occupy the ${{d}_{{{z}^2}}}$ and ${{d}_{{{x}^2} - {{y}^2}}}$ orbitals of the Mn ions equally and therefore no JT distortion is expected (Fig. 1c). However, for the (2, 4)-coordinated symmetry (Fig. S1), the elongated or shortened bond length facilitates the splitting of the *e_g_* and *t_2__g_* levels and Jahn–Teller distortion occurs (Fig. 1c).

**Figure 2.**
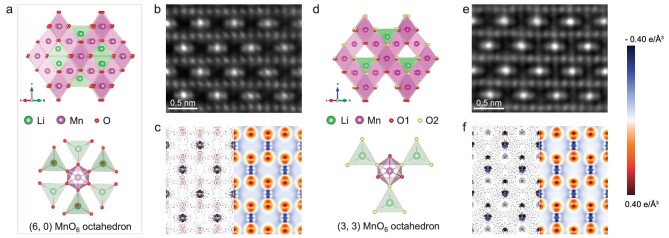
Atomic and electronic structures of LiMn_2_O_4_ and Li_0.5_Mn_2_O_4_. (a and d) Structure models of LiMn_2_O_4_ and Li_0.5_Mn_2_O_4_. The bottom panels show the symmetry of the MnO_6_ octahedron. (b and e) iDPC-STEM images of LiMn_2_O_4_ and Li_0.5_Mn_2_O_4_, respectively, along the [110] direction. (c and f) Real-space deformation electron density corresponding to panels (b) and (e), respectively. The left parts show the experimental results from multipole refinement and the right parts show the results from DFT calculations within the range of –0.4 ∼ 0.4 *e* Å^−3^. The contours interval is 0.1 *e* Å^−3^ in the experimental electron density map.

In the case of spinel LiMn_2_O_4_, the MnO_6_ octahedra share corners with LiO_4_ tetrahedra (see Fig. 2a), which are flexible, and the environments of the coordinated oxygen atoms can be changed via the Li-ion content. Specifically, in pristine LiMn_2_O_4_, each of the coordinated oxygen atoms of the MnO_6_ octahedron connects to a LiO_4_ tetrahedron, as shown in Fig. 2a, and all of the coordinated oxygen atoms are identical, which corresponds to the (6, 0) configuration. To obtain the (3, 3) configuration, we electrochemically extracted half of the Li ions from the spinel LiMn_2_O_4_. A rate of 0.1 C was used to ensure that the Li ions could be extracted homogeneously, such that each MnO_6_ octahedron could lose three corner-shared LiO_4_ tetrahedra (see Fig. 2d), resulting in a (3, 3)-configured Li_0.5_Mn_2_O_4_. We measured the length projections of the Mn–O bonds in the MnO_6_ octahedra along horizontal and vertical directions from the annular bright field scanning transmission electron microscopy (ABF-STEM) images of both LiMn_2_O_4_ and Li_0.5_Mn_2_O_4_, as shown in Fig. S2. There was no obvious change in the length projections of the Mn–O bonds. Besides the measurement of the local structure, synchrotron powder XRD (SPXRD) was employed to detect the macroscopic structure symmetry of LiMn_2_O_4_ and Li_0.5_Mn_2_O_4_ (Fig. 1d). Figures S3–S6 show that were are no diffraction peak splitting or new diffraction peaks in the two samples, indicating that there were no phase transitions and corresponding JT distortions. Besides the experiments, we calculated the density of states (DOS) of both structures through DFT using the Hubbard U corrected generalized gradient approximation (GGA + U) and the Heyd–Scuseria–Ernzerhof (HSE06) hybrid functional [30], respectively. Both the two total DOS of the Mn *3d* states and the partial DOS of the five individual *3d* orbitals in LiMn_2_O_4_ and Li_0.5_Mn_2_O_4_ from theoretical calculations (Figs S7 and S8) proved the energy degeneracy of the Mn *3d* orbitals. Especially in the case of HSE06, including a certain amount of Hartree–Fock exchange and the corrected self-interaction errors for metal and O atoms further improved the GGA results [31–33]. As shown in Fig. S8, the PDOS (projected density of states) of ${{d}_{{{z}^2}}}$ and ${{d}_{{{x}^2} - {{y}^2}}}$, as well as the PDOSs of ${{d}_{xy}}$, ${{d}_{xz}}$ and ${{d}_{yz}}$, coincide with each other, further verifying the degeneracy of the *e_g_* and *t_2__g_* orbitals of the Mn atoms in both LiMn_2_O_4_ and Li_0.5_Mn_2_O_4_.

To experimentally verify the degeneracy of the Mn *e_g_* orbital presented by using theoretical calculations, we performed multipole refinement over the combined structure factors obtained from QCBED and DFT calculations to yield a real-space 3D electron density distribution and to quantify the Mn *3d* orbital electron occupations of (6, 0)-LiMn_2_O_4_ and (3, 3)-Li_0.5_Mn_2_O_4_ (Note S1). We measured the first five low-order structure factors of LiMn_2_O_4_ and Li _0.5_Mn_2_O_4_, which were (111), (022), (113), (222) and (004), respectively. Tables S1 and S2 list the measured electron structure factors and the corresponding converted X-ray structure factors with the calculated X-ray structure factors from WIEN2k. Figure S9 exhibits the experimental measurement of the (111) and (222) structure factors of LiMn_2_O_4_ and Li_0.5_Mn_2_O_4_. Also, Table S3 lists all the structure factors used in the multipole refinement. To illustrate that the dose used in the CBED experiments would not have induced the atomic and electron structure changes of the sample, we performed electron diffraction and electron energy loss spectra experiments, as shown in Figs S10–S11 and Table S4. For detailed discussion, please see Note S2. The total density map is dominated by the core electrons, the deformation density, the difference between the measured aspherical electron density and the spherical atomic model, which clearly illustrates the redistribution of the valence electrons as a result of the bonding interactions [34]. The left panels of Fig. 2c and f show the experimental deformation electron densities of the Mn–O plane in the [110] direction for LiMn_2_O_4_ and Li_0.5_Mn_2_O_4_, respectively. The electron density map can be correlated with the corresponding atomic structure in Fig. 2b and e, which is visualized by using integrated differential phase contrast scanning transmission electron microscopy (iDPC-STEM),which is capable of directly imaging light and heavy elements [35]. The red and blue regions of the deformation density map represent the electron accumulation and depletion, respectively, under the ligand field. To validate the reliability of the experimental results, we performed theoretical calculations and compared the experimental and theoretical results. The right panels of Fig. 2c and f show the theoretical deformation electron densities of the same Mn–O plane. From the electron density distribution, it is clear that, in the (6, 0) configuration, the electron densities around the oxygen atoms are identical and, in the (3, 3) configuration, there are two types of electron density distributions around the oxygen atoms, which is consistent with the atomic model. This phenomenon can be seen also in Fig. S12. As a result, the Mn atoms are identical in both the (6, 0)- and (3, 3)-MnO_6_ configurations, indicating that no JTE appears.

In addition to the deformation densities, the occupations of the *t_2__g_* and *e_g_* orbitals of (6, 0)-LiMn_2_O_4_ and (3, 3)-Li_0.5_Mn_2_O_4_ can be quantified from the refined multipole population parameters, making the result more explicit. In multipole refinement, the deformation part of the valence electron density (the *d*-electron density for TM elements) at each atom can be modeled by using real spherical harmonics with multipole parameters. In the meantime, the *d*-orbital electron density can be described by using a linear combination of atomic orbitals, which also contain real spherical harmonics. The relationship between the refined multipole parameters and the *d*-orbital coefficients can be determined after normalizing the density functions and atomic orbitals. Therefore, the *d*-orbital electron occupations for TM atoms can be derived from the refined multipole parameters, as listed in Tables S5 and S6 [36]. As shown in Table 1, the measured number of electrons that occupy the Mn ${{d}_{{{z}^2}}}$ and ${{d}_{{{x}^2} - {{y}^2}}}$ orbitals are nearly the same for both (6, 0)-LiMn_2_O_4_ and (3, 3)-Li_0.5_Mn_2_O_4_, indicating the degeneracy of the *e_g_* orbitals of the Mn atoms. In addition, theoretically calculated orbital electron occupations using DFT + U and HSE06 of the Mn atoms are consistent with the experimental results. Therefore, both the experiments and the theoretical calculations confirm that the special symmetries of coordinate ligands can effectively suppress the JTE of MnO_6_ octahedra in manganese oxide materials. As such, we proposed a new local coordinate strategy to regulate the JTE of transition-metal octahedra at the orbital level.

**Table 1.** Mn *3d*-orbital electron occupations of LiMn_2_O_4_ and Li_0.5_Mn_2_O_4_.

	LiMn_2_O_4_			Li_0.5_Mn_2_O_4_		
Orbitals	Experiment	DFT + U	HSE06	Experiment	DFT + U	HSE06
*P_v_*	4.7387	4.9026	4.7662	4.5941	4.6756	4.7524
${{d}_{{{z}^2}}}$	0.6993 (141)	0.8427	0.8772	0.6990 (374)	0.8185	0.8631
${{d}_{{{x}^2} - {{y}^2}}}$	0.7013 (123)	0.8246	0.8772	0.6929 (351)	0.8134	0.8631
${{d}_{xy}}$	1.1804 (123)	1.2207	1.0040	1.1154 (351)	1.0139	1.0087
${{d}_{xz}}$	1.0805 (127)	1.0075	1.0040	1.0268 (360)	1.0148	1.0087
${{d}_{yz}}$	1.1285 (127)	1.0071	1.0040	1.0691 (360)	1.0150	1.0087

## CONTROLLING THE JAHN–TELLER DISTORTION IN Mn-BASED OXIDES

**Figure 3.**
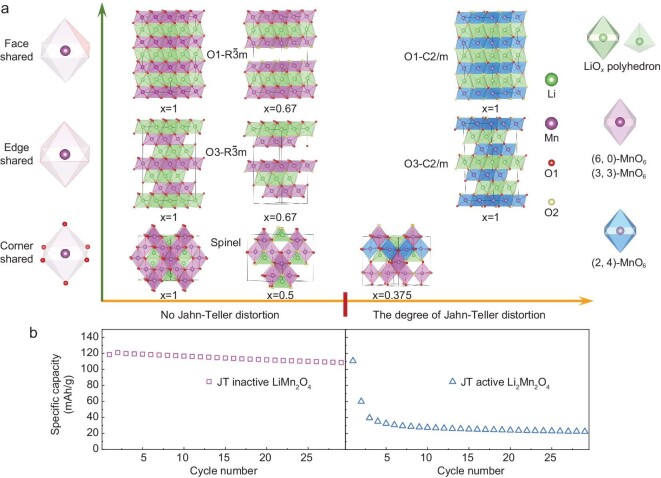
The way to control the JTE by regulating the ligand symmetries of the MnO_6_ octahedra for Mn-based cathodes. (a) The correlation between the symmetry of coordinated oxygen atoms and the JTE. The configurations containing the (6, 0), (3, 3) and (2, 4) symmetries were constructed by sharing the corners, edges and faces of the MnO_6_ octahedra with the adjacent LiO*_x_* polyhedra. (b) The capacity retention of LiMn_2_O_4_ (JT inactive with (6, 0) symmetry) at 3.5–4.2 V and Li_2_Mn_2_O_4_ (JT active with (2, 4) symmetry) at 2.5–3.2 V. No JTE observably improves the structural stability during electrochemical cycling.

To further verify the effectiveness of the proposed local coordinate strategy for controlling the JTE of manganese oxide cathodes, we adopted different connection modes between the MnO_6_ octahedra and the adjacent LiO*_x_* polyhedra to tune the symmetry of the coordinated oxygen atoms containing the (6, 0), (3, 3) and (2, 4) symmetries. As shown in Fig. 3a, the MnO_6_ octahedron in O1-Li_x_MnO_2_ ($R\bar{3}m$), O3-Li_x_MnO_2_ ($R\bar{3}m$) and spinel Li_x_Mn_2_O_4_ shares faces, edges and corners with the adjacent LiO*_x_* polyhedra, respectively. All of these structures with *x* = 1 contain the (6, 0) symmetry. There are (6, 0) and (3, 3) symmetries in O1- and O3-Li_0.67_MnO_2_ ($R\bar{3}m$), as well as spinel Li_0.5_Mn_2_O_4_. Both (2, 4) and (3, 3) symmetries exist in spinel Li_0.375_Mn_2_O_4_. Only (2, 4) symmetry exists in O1- and O3-LiMnO_2_ (*C2/m*). DFT calculations demonstrate that all structures containing only (6, 0) and (3, 3) symmetries are free of the JTE, while structures with (2, 4) symmetry have the JTE. It is clear that, regardless of what the MnO_6_ octahedra share with the adjacent LiO*_x_* polyhedra, as long as the condition of (6, 0) and (3, 3) symmetries is satisfied, the JTE is suppressed (Fig. 3a). In addition, Fig. 3b shows the capacity retention of LiMn_2_O_4_ (JT inactive with (6, 0) symmetry) at 3.5–4.2 V and Li_2_Mn_2_O_4_ (JT active with (2, 4) symmetry) at 2.5–3.2 V, revealing the dramatic difference in the structural stability between JT inactive and JT active configurations.

**Figure 4.**
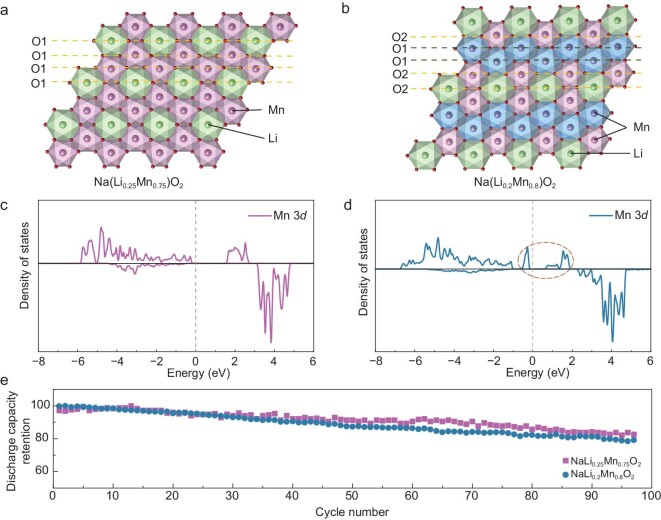
Electron structure and electrochemistry of honeycomb- and ribbon-ordered Na(Li*_x_*Mn_1__–_*_x_*)O_2_ (*x* = 0.25 and 0.2). (a and b) The ordering structures of the transition-metal layer along the [001] direction. The honeycomb-ordering NaLi_0.25_Mn_0.75_O_2_ corresponds to a (6, 0) configuration. The ribbon-ordering NaLi_0.2_Mn_0.8_O_2_ corresponds to an atypical (2, 4) symmetry. (c and d) The PDOS of the Mn *3d* state in NaLi_0.25_Mn_0.75_O_2_ and NaLi_0.2_Mn_0.8_O_2_, respectively. In the (6, 0)-NaLi_0.25_Mn_0.75_O_2_, all the Mn atoms are equivalent, so we arbitrarily chose one Mn atom to show its *3d*-state PDOS. In the (2, 4)-NaLi_0.2_Mn_0.8_O_2_, we chose a Mn atom in the blue octahedra, which is a JT distorted octahedron. (e) The discharge capacity retention of NaLi_0.25_Mn_0.75_O_2_ and NaLi_0.2_Mn_0.8_O_2_, respectively, cycled under a voltage of 2.5–1.5 V. The first two cycles were under a current rate of 0.1 C and the subsequent cycles were under a current rate of 0.5 C.

Besides LIB Mn-based cathode materials, we applied the strategy on sodium-ion battery Mn-based cathodes and demonstrated its availability. As seen in Fig. 4, we constructed and synthesized two ordering structures: honeycomb-ordered NaLi_0.25_Mn_0.75_O_2_ with (6, 0) configuration and ribbon-orderd NaLi_0.2_Mn_0.8_O_2_ with atypical (2, 4) configuration, respectively. In the honeycomb-ordered NaLi_0.25_Mn_0.75_O_2_ (Fig. 4a), all ligand oxygens in the MnO_6_ octahedra connected with the other two Mn atoms and one Li atom, leading to a (6, 0) configuration, and the total Mn–O bond length is 1.96 Å. Therefore, there is no JT distortion in this structure. Although this is not the ideal Li/Mn ratio, it is close to that of the ribbon-orderd NaLi_0.2_Mn_0.8_O_2_, so that the valence states of the Mn in the two complexes are very close to each other. In the ribbon-ordered NaLi_0.2_Mn_0.8_O_2_, ligand oxygens in the MnO_6_ octahedra are divided into two kinds according to the connection types. As shown in Fig. 4b, each O1 connects with one Mn atom and two Li atoms, and each O2 connects with two Mn atoms and one Li atom, leading to an atypical (2, 4) configuration. As a result, the MnO_6_ octahedra distort to reduce the energy of the system. According to the symmetry of the ligand oxygens, the MnO_6_ octahedron is divided into two classes, which are colored using blue and purple, respectively. The calculated Mn–O bond lengths in the above two kinds of MnO_6_ octahedra are listed in Table S7. Since the valence states of the Mn in NaLi_0.25_Mn_0.75_O_2_ and NaLi_0.2_Mn_0.8_O_2_ are 3.67+ and 3.5+, respectively, which are very close to each other, the occurrence of JT distortion is only related to the configuration of the MnO_6_ octahedron and has nothing to do with the valence state. We performed XRD to verify the structure of the synthesized Na(Li*_x_*Mn_1__–_*_x_*)O_2_. A detailed discussion is shown in the information. Because the ordering structure is difficult to refine, the space group P6_3/mmc_ is generally chosen to be the model to refine the structure. In addition, we calculated the DOS of the Mn *3d* state for the above two structures. In (6, 0)-NaLi_0.25_Mn_0.75_O_2_, the uniform distribution of the Mn *3d* states near the Fermi level indicates that the electrons occupy the orbitals evenly, with no JT distortion occurring (Fig. 4c). In contrast, in (2, 4)-NaLi_0.2_Mn_0.8_O_2_, the presence of a sharp peak in the Mn *3d* states near the Fermi level indicates orbital splitting, signifying that JT distortion is occurring (Fig. 4d). To verify the influence of JT distortion on the structural stability of the cathode materials, we compared the discharge capacity retention of the two above compounds, which is related to the structural stability, as can be seen in Fig. 4e. In order to find the relationship between the JTE and the structural stability of the cathode materials, we set the cycling range of 1.5–2.5 V, which ruled out the influence of the oxygen redox. Although there is little difference between the two cathode materials, the structural stability of (6, 0)-NaLi_0.25_Mn_0.75_O_2_ is superior to that of (2, 4)-NaLi_0.2_Mn_0.8_O_2_.

## CONTROLLING THE JAHN–TELLER DISTORTION IN TRANSITION-METAL COMPOUNDS

**Figure 5.**
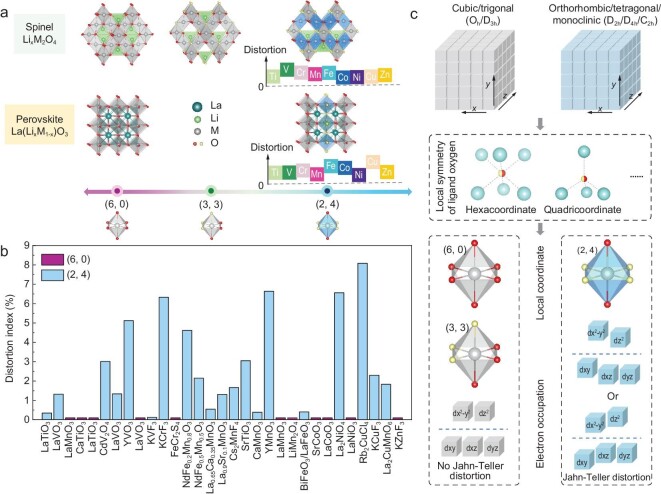
Regulation of the JTE for the spinel- and perovskite-3*d* transition-metal oxides. (a) Constructed structures with different local coordinate symmetries. The symmetry of the MO_6_ octahedron is (6, 0) in cubic spinel LiM_2_O_4_ and perovskite LaMO_3_, and (3, 3) in Li_0.5_M_2_O_4_. The (2, 4) symmetry of the MO_6_ octahedron appears in JT distorted Li_0.5_M_2_O_4_ and La(Li_1/6_M_5/6_)O_3_. The gray octahedra represent the MO_6_ (M = Ti, V, Cr, Mn, Fe, Co, Ni, Cu and Zn) octahedra in the spinel and perovskite frameworks. The degrees of distortion of the (2, 4)-MO_6_ octahedra are illustrated at the bottom of the (2, 4) configurations for the spinel and perovskite structures. (b) Experimental statistic results according to the classification of the local coordinate. The distortion indexes are actually zero for (6, 0) and (3, 3) configurations, which are set as 0.1 in the graph for comparison. The definition of the distortion index is illustrated in Tables S9 and S10. (c) The correlation between the macroscopic symmetry of phase, local atomic structure and electronic structure for regulating the JTE. The cyan spheres represent TM and alkali-metal ions.

Because the JTE is always accompanied by lattice distortion, this leads to different electronic states, such as charge ordering, orbital ordering and spin ordering. Accordingly, controlling the JT distortion in TM oxides using the above strategy will induce divergent physical properties. In addition to spinel compounds, perovskite ABX_3_ is an ideal framework because the cubic lattice structure favors the electronic degeneracy of the B-site TM ions, which can, in turn, be lifted via a lattice distortion and therefore induce multiple intriguing phenomena. As such, we chose LiM_2_O_4_ and LaMO_3_ (M = Ti, V, Cr, Mn, Fe, Co, Ni, Cu and Zn) as the prototypes to regulate the ligand symmetry of the MO_6_ octahedron according to our local strategy by extracting the Li ions from LiM_2_O_4_ and replacing M with Li atoms in LaMO_3_. The reason why we substituted Li for M is that the weak bonding interaction between Li and O can lengthen the Li–O distance and change the ligand configurations of the adjacent MO_6_ octahedra. We then investigated the type of JT distortions of the MO_6_ octahedron using DFT + U calculations. Tables S8 and S9 list the M–O bond lengths and degrees of distortion of the MO_6_ octahedra induced by changing the ligand symmetry of the spinel and perovskite frameworks, respectively. Figure 5a shows the (6, 0), (3, 3) and (2, 4) configurations in spinel LiM_2_O_4_ and perovskite La(Li*_x_*M_1__–_*_x_*)O_3_. There are no JT distortions for the (6, 0) and (3, 3) symmetries. The JT distortion only exists in the (2, 4) configurations in the spinel and perovskite structures. We also find that, once the (2, 4) symmetry is satisfied, the JT distortion occurs for the MO_6_ octahedron, regardless of TM ions. The different degrees of distortion only rely on the local chemical environments and polyhedra adjacent to the MO_6_ octahedron.

Besides, we also analysed the local coordination symmetries of some spinel and perovskite compounds containing the MX_6_ (X = O, F, S and Cl) octahedra from experimental data and proved the reliability of the orbital strategy to control the JTE based on the local coordinate symmetries. Figure 5b shows the distortion of the MX_6_ octahedra based on the bond length (see Tables S10 and S11). We summarized the macroscopic symmetries corresponding to the local symmetries of oxygen in the crystal structure from Fig. 5b. It was observed that cubic and trigonal structures correspond to the (6, 0) and (3, 3) configurations. Orthorhombic, tetragonal and monoclinic structures correspond to the (2, 4) configuration. The corresponding point groups are also illustrated. Figure 5c indicates that, in crystals with specific macroscopic symmetries, there are differences in the local symmetry of oxygen, leading to the mentioned (6, 0), (3, 3) and (2, 4) configurations. This results in changes in the degeneracy of the electron orbital energy levels and electron redistribution, accompanied by Jahn–Teller distortions. In summary, we established correlations between the macroscopic symmetry of phase, local atomic structure and electron structure to control Jahn–Teller distortions, guiding material design. On the basis of the above results and findings, it was found that the proposed local coordinate strategy that originated from the orbital level for regulating the JTE is more universal than other macroscopic strategies. Through changing the ligand symmetry of the MX_6_ octahedron, one can control the JT distortion and corresponding functional electronic properties. This can be achieved via temperature changes, strain engineering and elemental substitution [37,38].

## CONCLUSION

In summary, because of the important role played by the JTE in affecting the physical properties of TM oxides via the lattice, charge, orbital and spin degrees of freedom, controlling the JTE and the corresponding electronic structures is highly desirable to control and design the functionalities of materials. Using LiMn_2_O_4_ and Li_0.5_Mn_2_O_4_ as prototypes, we measured the Mn *3d*-orbital electron occupations via QCBED and found that the number of electrons in the Mn ${{d}_{{{z}^2}}}$ and ${{d}_{{{x}^2} - {{y}^2}}}$ orbitals were nearly the same, demonstrating that there is no JTE in both samples at the orbital level. By analysing the symmetry of the coordinated oxygen atoms in the MnO_6_ octahedron, we found that the (6, 0)- and (3, 3)-MnO_6_ configurations can maintain Mn *e_g_* orbital degeneracy, therefore effectively suppressing JT distortion. Based on the above findings, we proposed a new local coordinate strategy to control the JTE. This strategy, on the basis of the direct relationship between orbital and local coordinate symmetry, is universal for oxide functional materials possessing MO_6_ octahedra. To verify the effectiveness of this orbital strategy, we applied it to P2-type Na(Li*_x_*Mn_1__–_*_x_*)O_2_ (*x* = 0.25 and 0.2) and the spinel and perovskite frameworks containing the MX_6_ octahedra with different coordinate symmetries and proved the universality of it. These findings provide a universal orbital engineering strategy to regulate JT distortion and should inspire ongoing efforts toward designing structures with divergent physical properties for functional materials.

## METHODS

### Sample preparation

Pristine LiMn_2_O_4_ powder with a purity of 99.5% was purchased from Alfa. High-loading electrodes (∼100 mg) were charged to various states of charge (delithiated samples) in a Swagelok cell with lithium as the counter electrode, using 1 M LiPF_6_ in ethylene carbonate and dimethyl carbonate as the electrolyte. The charged battery was disassembled and the obtained powder sample was washed three times with dimethyl carbonate, followed by drying. All the steps were conducted in an argon-filled glove box. The preparation of NaLi_0.25_Mn_0.75_O_2_ and NaLi_0.2_Mn_0.8_O_2_ included two steps. In the first step, MnO_2_ (99.9% Alfa), Na_2_CO_3_ (99.9% Alfa) and LiOH (98% Alfa) were used as precursors. The mixture was heated at 900°C for 15 hours in an air atmosphere with a controlled heating rate of 5°C/min and a cooling rate of 2°C/min. Then, the products were discharged to 1.5 V to prepare the final samples.

### QCBED collection and SPXRD measurements

The QCBED experiments were performed using a FEI Tecnai G2 F20 S-TWIN transmission electron microscope equipped with a Gatan imaging filter and 1024 × 1024-pixel charge-coupled device camera. Synchrotron powder X-ray profiles were measured at a SPring-8 BL19B2 beamline. See Note S1 for details.

### STEM characterization

Atomic-resolution iDPC-STEM images were acquired on a Thermo-Fisher Titan Cubed Themis G2 electron microscope equipped with a DCOR+ corrector. The electron beam acceleration voltage was 300 kV, with a convergence semi-angle of 10 mrad [39]. A high-pass filter was applied to the atomic-resolution images to filter out low-frequency signals and enhance the signal-to-noise ratio. Four images were acquired using a quadrant DF4 detector for 2D integration. Annular bright field scanning transmission electron microscopy imaging was conducted using an aberration-corrected electron microscope (JEOL-ARM200CF) operating at 200 kV, with a convergence angle of 28 mrad and a collection angle of 13–24 mrad.

### Electron energy loss spectroscopy spectra

Electron energy loss spectroscopy spectra were obtained in STEM mode using a 200-kV aberration-corrected JEOL-ARM200CF microscope. The probe current was 120 pA, with an energy dispersion of 0.25 eV/ch and an exposure time of 0.01 seconds per pixel.

### Theoretical calculations

The Vienna Ab Initio Simulation Package (VASP) [40] and WIEN2k [41] were used to calculate the atom and electron structures. See Note S3 for details.

## Supplementary Material

nwae255_Supplemental_File
