# Supplementary material for: An orbital strategy for regulating the Jahn–Teller effect
Source: Natl Sci Rev. 2024 Aug 5;11(9):nwae255. doi: 10.1093/nsr/nwae255 (PMC11339606; doi:10.1093/nsr/nwae255)
Supplement: nwae255_Supplemental_File [file nwae255_supplemental_file.pdf]

Supplementary Information

An orbital strategy for regulating the Jahn–Teller effects

Tongtong Shang[1,2,]†, Ang Gao[1,2,]†, Dongdong Xiao[2, 3,]†, Qinghua Zhang[2,]†, Xiaohui Rong[2,]†, Zhexin Tang[2], Weiguang Lin[2], Ting Lin[2], Fanqi Meng[1], Xinyan Li[2], Yuren Wen[4], Xuefeng Wang[2], Dong Su[2], Zhen Chen[1], Yong-Sheng Hu[2], Hong Li[2], Qian Yu[5], Ze Zhang[5], Lijun Wu[6,]*, Lin Gu[1,2,]*, Jian-Min Zuo[7], Yimei Zhu[6], Liquan Chen[2] and Ce-Wen Nan[1,]*

[1]State Key Laboratory of New Ceramics and Fine Processing, National Center for Electron Microscopy in Beijing, School of Materials Science and Engineering, Tsinghua University, Beijing 100084, China;

[2]Beijing National Laboratory for Condensed Matter Physics, Institute of Physics, Chinese Academy of Sciences, Beijing 100190, China;

[3]Songshan Lake Materials Laboratory, Dongguan 523808, China;

[4]School of Materials Science and Engineering, University of Science and Technology Beijing, Beijing 100083, China;

[5]Department of Materials Science and Engineering, Center of Electron Microscopy and State Key Laboratory of Silicon Materials, Zhejiang University, Hangzhou 310027, China;

[6]Condensed Matter Physics and Materials Science Division, Brookhaven National Laboratory, New York (NY) 11973, USA

[7]Department of Materials Science and Engineering, University of Illinois at Urbana Champaign, Urbana (IL), 61801, USA

***Corresponding authors.** E-mails: ljwu@bnl.gov; lingu@mail.tsinghua.edu.cn; cwnan@mail.tsinghua.edu.cn

†Equally contributed to this work.

Supplementary Note

1. Mapping the electron density and *d*-orbital populations

QCBED collection. The QCBED experiments were performed using a FEI Tecnai G2 F20 S-TWIN transmission electron microscope equipped with a Gatan imaging filter and 1024 × 1024-pixel charge-coupled device camera. Near the zero-loss peak, select an energy window of 10 eV. Due to the use of energy filters in QCBED experiments, inelastic scattered electrons are excluded from the Bloch wave calculations. Two convergence angles were used to obtain the systematic row patterns depending on the different d-spacings of the sample. The accelerating voltage was precisely measured at 198.50 kV through fitting the QCBED pattern from dynamic simulations with experimental patterns obtained from single-crystal silicon samples.

SPXRD measurements. Polycrystalline samples of $LiMn_2O_4$ and $Li_{0.5}Mn_2O_4$ were sealed in a Lindeman glass capillary with an internal diameter of 0.3 mm. Synchrotron powder X-ray profiles were measured at a SPring-8 BL19B2 beamline. A large Debye–Sherrer camera with an imaging plate detector was used for data collection. Data was collected at room temperature. The wavelength of the incident X-rays was 0.41334 Å, as calibrated by an NIST CeO_2 standard sample. All data were collected with sin $\theta/\lambda max = 1.67$ Å^{-1}.

Before performing the multipole refinement, accurate structure factors are needed. Quantitative CBED was implemented to accurately measure the low-order crystal structure factors that are sensitive to valence electrons, as illustrated in **Fig. S9,** where structure factor measurements were made by comparing experimental intensity profiles across QCBED disks (rocking curves) with calculations, using a goodness-of-fit (GOF) criterion[1]. **Tables S1** and **S2** show the refined and converted structure factors. Considering that low temperature can induce phase transition of the spinel $LiMn_2O_4$, our QCBED experiments were performed at 300K (room temperature). Since battery materials are highly sensitive to electron beam damage, we carefully optimized the experimental conditions for QCBED to avoid the beam damage. Both the CBED patterns and electron energy loss spectra indicate that the electron dose used in our experiment did not induce atomic and electronic structural changes (see **Figs. S10-11**, **Table S4**). Since the high-order structure factors are primarily determined by the atomic position and core electrons, X-ray diffraction or DFT calculations can be employed to complement the higher-order structure factors without losing significant accuracy. In this study, the higher-order structure factors were calculated using WIEN2K[2] with a fully-potential linear augmented plane-wave method[3], that can guarantee highly accurate results. After

acquiring the QCBED pattern of $Li_{1-x}Mn_2O_4$, we used a to refine the low-order structure factors[1]. The refined parameters include the electron-beam incident direction, the sample thickness, and the structure factors. To avoid very large matrices and a reasonable accuracy for the Bloch wave theory, electron beams were selected according to certain criteria[4]. 2.6 for g_{max}, 3.0 for $2KS_{gmax}$, 0.005 for $\left|\frac{U_{max}}{2KS_g}\right|$ were adopted for refinement, respectively.

Multipole refinement provides the most efficient parameterization of the real-space charge density and gives a result that is insensitive to missing reflections in the collected dataset[5, 6]. In this method, the crystal charge density is fitted by a sum of non-spherical pseudo-atomic densities. With the refined population parameters for real spherical harmonics in the valence part, one can determine the charge transfer and rearrangement of orbital due to the chemical bonding and the local symmetries[5, 7]. To perform the multipole refinement, we need to convert the refined electron structure factors to the X-ray structure factors through the Mott-Bethe formula. The refined low-order electron and converted X-ray structure factors are listed in **Table S1–S2** for $LiMn_2O_4$ and $Li_{0.5}Mn_2O_4$, respectively. In this work, the high-order structure factors are obtained from the DFT calculations using the full potential linear augmented plane wave method implemented in WIEN2K[2].

The multipole refinements were performed in JANA2006 software[8]. As shown in **Eq. S1,** the multipole model is based on the real spherical harmonics which centered around each atomic nucleus[5, 9]. As to d electrons, the l_{max} = 4 at the hexadecapole level is enough. The coordinate systems used for both Mn ($\bar{3}m$) and O ($3m$) are that the z-axis is parallel to the 3-fold axis $[111]$ direction, and the y axis is perpendicular to the mirror plane, i.e., alone $[1\bar{1}0]$ direction. Several constraints were imposed on the multipole refinement: the valence population of Li ion was fixed at P_v = 0, $\kappa_{[Li]}$ = 1 and the deformation electron density of Li was not refined. For Mn, the two *4s* electrons were treated as core electrons, and the five *3d* electrons were considered as valence electrons[10].

$$\rho(\boldsymbol{r}) = \rho_c + P_v \kappa^3 \rho_v(\kappa r) + \sum_{l=0}^{l_{max}} \kappa'^3 R_l(\kappa' r) \sum_{m=0}^{l} P_{lm\pm}\, d_{lm\pm}(\theta, \phi) \qquad \text{(S1)}$$

P_v is the valence electron population parameter. $d_{lm\pm}$ is real spherical harmonics with the population parameter $P_{lm\pm}$. The κ and κ' parameters characterize the radial expansion-contraction of the valence electron density for the spherical and aspherical part, respectively. R_l is the radial function.

2. Electron dose and beam damage

Cathode materials are very sensitive to the electron beam. To avoid and reduce the beam damage, when collecting the diffraction patterns, we continually keep moving the sample and grasping as fast as possible to reduce the recording time. Due to the different *d*-spacings, we collected the CBED data with two different condenser lens (CL) apertures while keeping other conditions unchanged. For (111) and (222), the convergent semi-angle of 3.1 mrad was adapted. For other three structure factors, the convergent semi-angle of 4.3 mrad was adapted. All the experimental conditions were the same for $LiMn_2O_4$ and $Li_{0.5}Mn_2O_4$, including the convergent semi-angles. **Table S4** presents the beam currents and electron doses of the above mentioned two experimental conditions.

As the beam damage could induce the change of atomic and electronic structures, we performed electron diffraction and electron energy loss spectroscopy to verify that the dose used in the QCBED experiments is under the safe range. In general, compared with parallel beam electron diffraction, the diffraction discs of CBED contain rich structural information, and therefore are more sensitive to structural changes[11]. **Fig. S10** exhibits some CBED patterns of $LiMn_2O_4$, which clearly show the changes of the contrast before and after beam damage. We selected the CBED patterns without any fringes and HOLZ line splitting during refining the structure factors. In addition, due to the high sensitivity of the CBED pattern to structural changes, defects induced by beam damage will lead to a large deviation between the refinement results and the experimental intensity.

Besides, STEM-EELS experiments of the specimen were performed to detect the electronic structure changes. The electron doses used in CBED collection were too small to obtain adequate signal to noise ratio in TEM-EELS. Thus, we performed STEM-EELS to evaluate the electronic structure changes during the electron beam irradiation. The beam current is 120 pA, which is equal to the largest beam current used in CBED. To simulate the situations in TEM mode, we set a square area with 0.308×0.308 nm, and adjust the defocus slightly. In this case, the electron beam did not focus on the sample, and has almost the same diameter with the irradiated area. The pixels of the selected irradiated area were divided into 30×30, and the exposure time was 0.01 s. Each 10 spectra were superposed to obtain an EELS spectrum collected with 0.1 s. **Fig. S11** illustrates the EELS spectra of $LiMn_2O_4$ and $Li_{0.5}Mn_2O_4$ and shows that the electronic structures start to change slightly at 0.3 s for both samples, indicating that the electron dose used in CBED collection (exposure time of 0.1 s) would not induce the obvious electronic-structure changes of the samples.

3. DFT calculations

The Vienna Ab Initio Simulation Package (VASP) based on the DFT was used to calculate the electron density and electronic structures of $LiMn_2O_4$, $Li_{0.5}Mn_2O_4$, layered-$LiMnO_2$, layered-$Li_{0.67}MnO_2$, layered-$Li_{0.5}MnO_2$, spinel $Li_{1-x}MO_2$ and perovskite $La(Li_xM_{1-x})O_3$. The Perdew–Burke–Ernzerhof (PBE) functional within a generalized gradient approximation (GGA) form was adopted to treat the exchange–correlation energy. DFT+U was used to correct the self-interaction error of conventional DFT for correlated *d* electrons. U values of 3.25, 3.7, 3.9, 5.3, 3.32, and 6.2 eV were used for V, Cr, Mn, Fe, Co, and Ni, respectively. A plane wave representation for the wave function with a cut off energy of 500 eV was applied. Geometric optimizations were performed using conjugate gradient minimization until all forces acting on the ions were less than 0.01 eV/Å per atom. The K-point mesh with a spacing of *ca.* 0.03 $Å^{-1}$ was adopted. The extended supercells of spinel-$Li_8M_{16}O_{32}$, layered-$Li_{12}Mn_{12}O_{24}$, and perovskite-$La_{12}(Li_2M_{10})O_{36}$ were adopted for the geometric optimizations and electronic structures (where M indicates a *3d* TM). Beside DFT+U, we also adopted HSE06 on $LiMn_2O_4$ and $Li_{0.5}Mn_2O_4$ with the standard mixing parameter ($\alpha = 0.25$) to verify the results from DFT+U calculation and the experiment. WIEN2k using a fully-potential linear augmented plane-wave method was adopted to obtain the structure factors of $LiMn_2O_4$ and $Li_{0.5}Mn_2O_4$ *via* Fourier transformation of the theoretical electron density. The input structure of $LiMn_2O_4$ was set as refined from experimental data, while the optimized structure from VASP was used for $Li_{0.5}Mn_2O_4$. In both cases, the PBE-GGA was adopted as the exchange-correlation functionals, and the select energy and the energy convergence criterion were set to be -6.0 Ry and 10^{-5} eV respectively.

To consolidate the reliability of our mechanism, we constructed several configurations of manganese-based oxide cathodes, spinel- and perovskite-type *3d* transition-metal oxides with different coordinated oxygen symmetries, and analyzed the octahedron distortion and electronic structures of the corresponding M (from Ti to Zn) atoms. In order to regulate the coordinated oxygen symmetries, we select spinel $LiMn_2O_4$, O3-layered $LiMnO_2$ and O1-layered $LiMnO_2$, they share corners, edges and faces with the adjacent LiO_x polyhedron, respectively. Removing the Li atoms from the above structures leads to form the (6, 0), (3, 3), and (2, 4) configurations in these structures. For the spinel $Li_{1-x}M_2O_4$ framework, the constructed structure is the same as the $Li_{1-x}Mn_2O_4$. For the perovskite $LaMO_3$ framework, we replace 1/6 of the M atoms by the Li atom to construct the (2, 4)-MO_6 octahedrons. After constructing the structures, we calculate the electronic structures within the GGA+U scheme, with the projector augmented wave

method[12] as implemented in the VASP[13, 14]. The atom positions are relaxed with the conjugated gradient method, and the final force on each atom is less than 0.005 eV/Å.

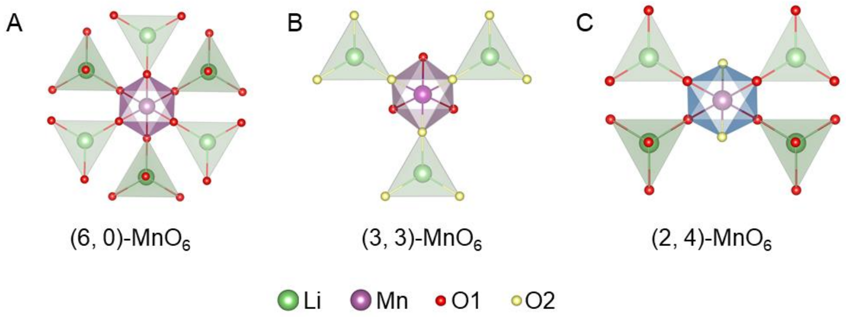

Fig. S1. Three typical MnO_6 octahedra with different ligand symmetries.

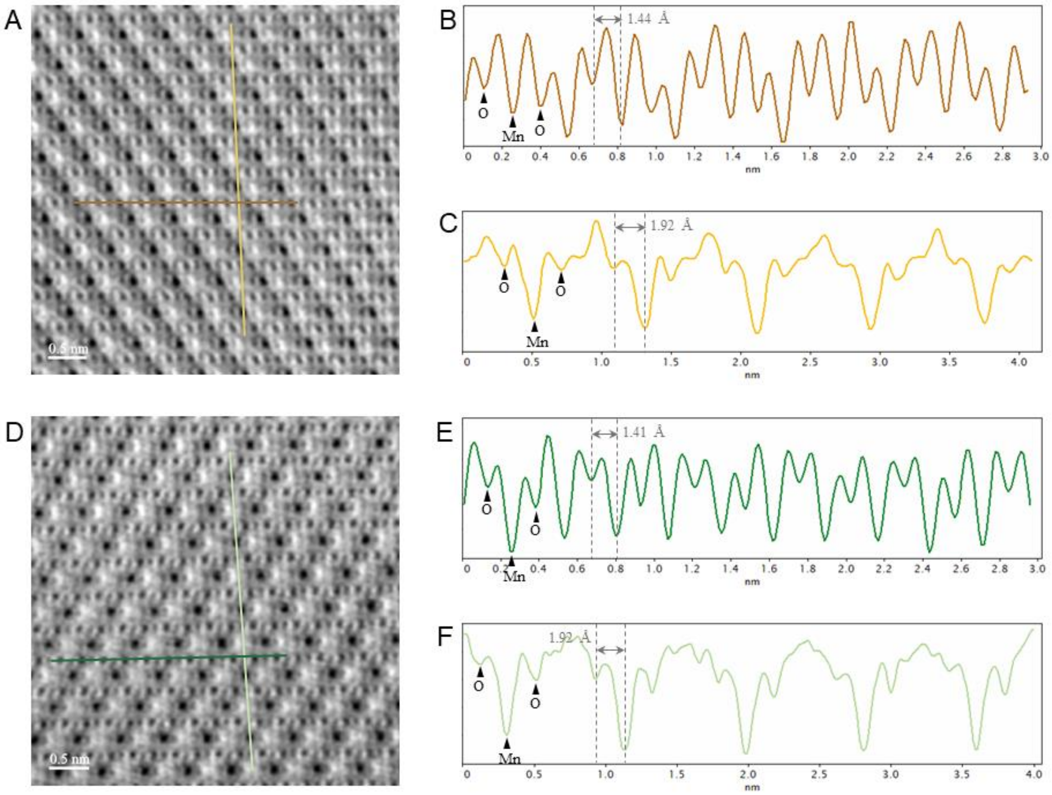

Fig. S2. The length projections of the Mn–O bond in MnO_6 octahedra for $LiMn_2O_4$ and $Li_{0.5}Mn_2O_4$. (A) The STEM-ABF image of $LiMn_2O_4$ along the [110] direction. The inside dark-yellow line and light-yellow line correspond to the projection of the Mn–O bond along (B) horizontal and (C) vertical directions. (D) The STEM-ABF image of $Li_{0.5}Mn_2O_4$ along the [110] direction. The inside dark-green line and light-green line correspond to the projection of the Mn–O bond along (E) horizontal and (F) vertical directions.

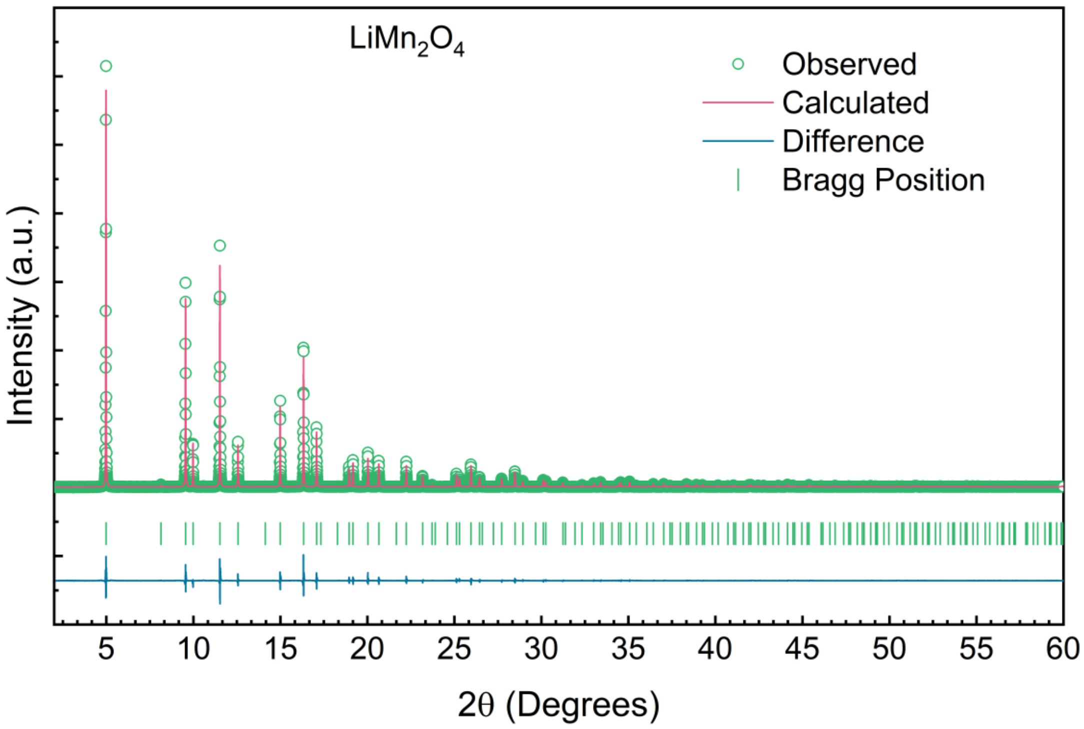

Fig. S3. Rietveld refinement for the SPXRD pattern of $LiMn_2O_4$.

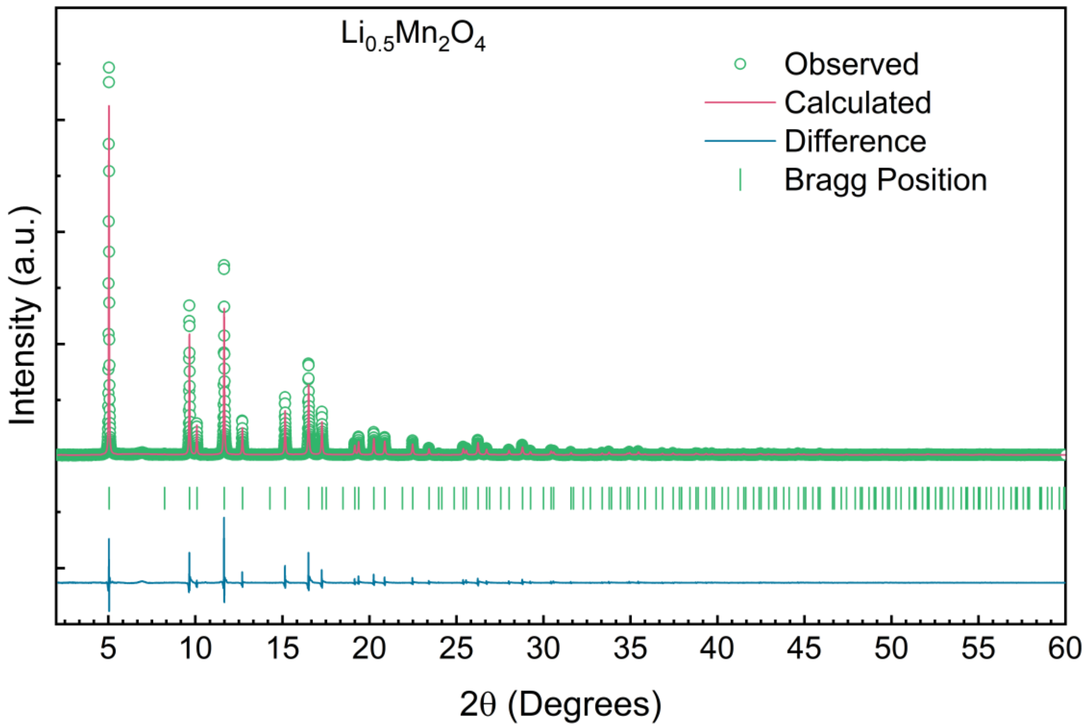

Fig. S4. Rietveld refinement for the SPXRD pattern of $Li_{0.5}Mn_2O_4$.

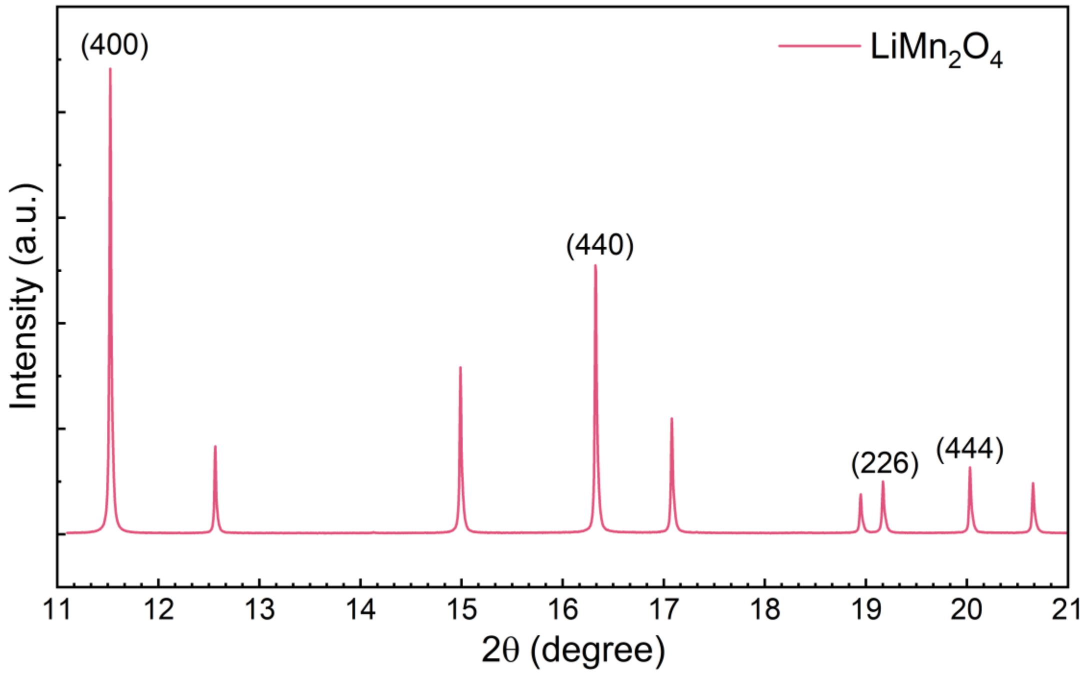

Fig. S5. Expanded synchrotron powder X-ray diffraction pattern for $LiMn_2O_4$. No splitting of diffraction peaks indicates there is no phase transition from the cubic to tetragonal structure.

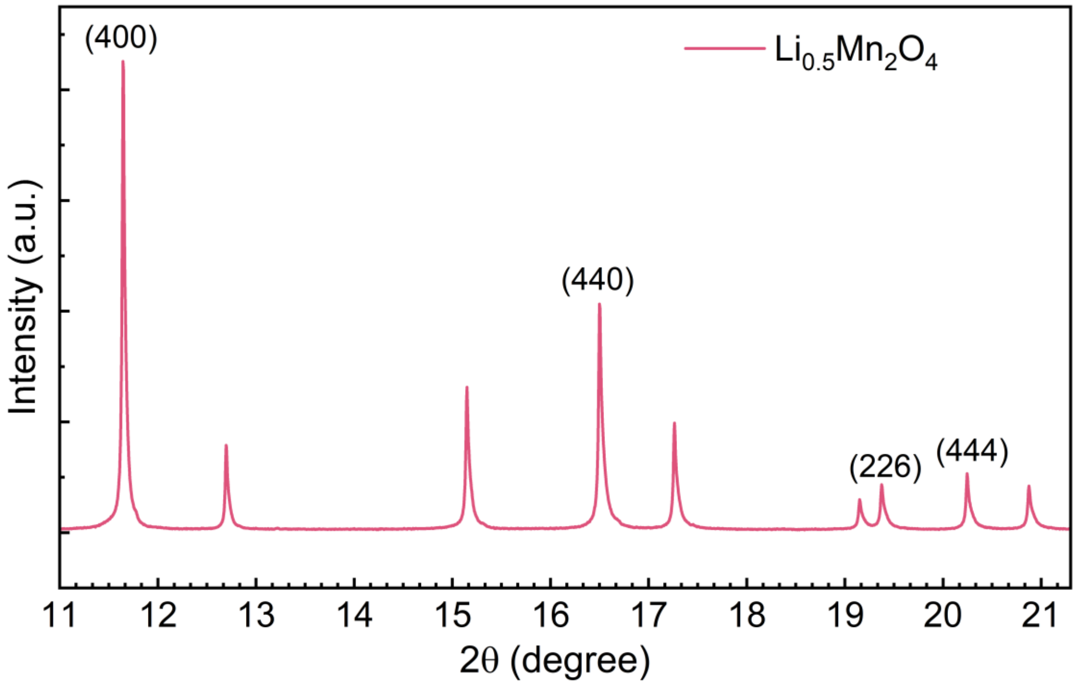

Fig. S6. Expanded synchrotron powder X-ray diffraction pattern for $Li_{0.5}Mn_2O_4$. No splitting of diffraction peaks indicates there is no phase transition from the cubic to tetragonal structure.

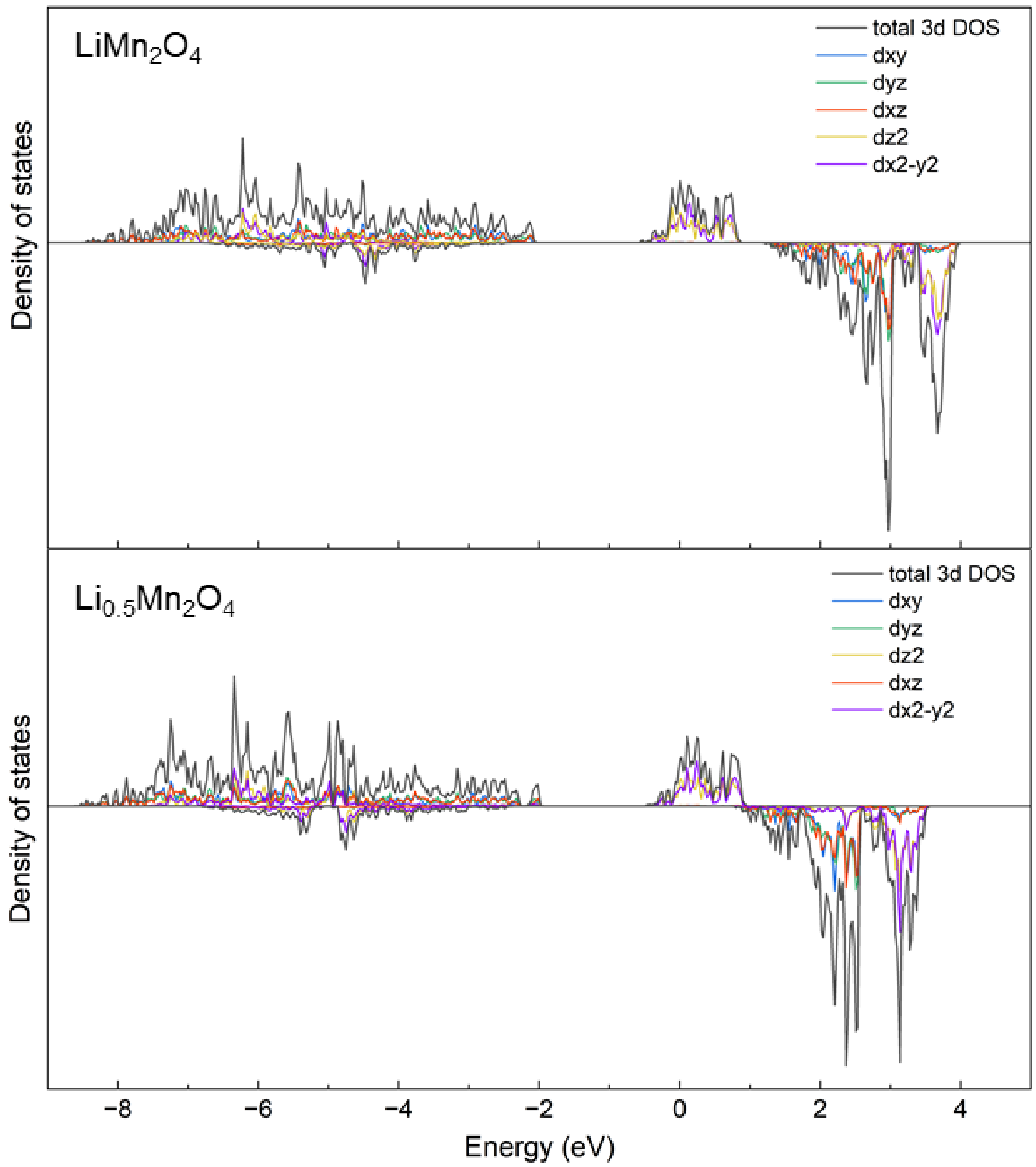

Fig. S7. PDOS of Mn *3d* state of $LiMn_2O_4$ and $Li_{0.5}Mn_2O_4$ using DFT+U, respectively.

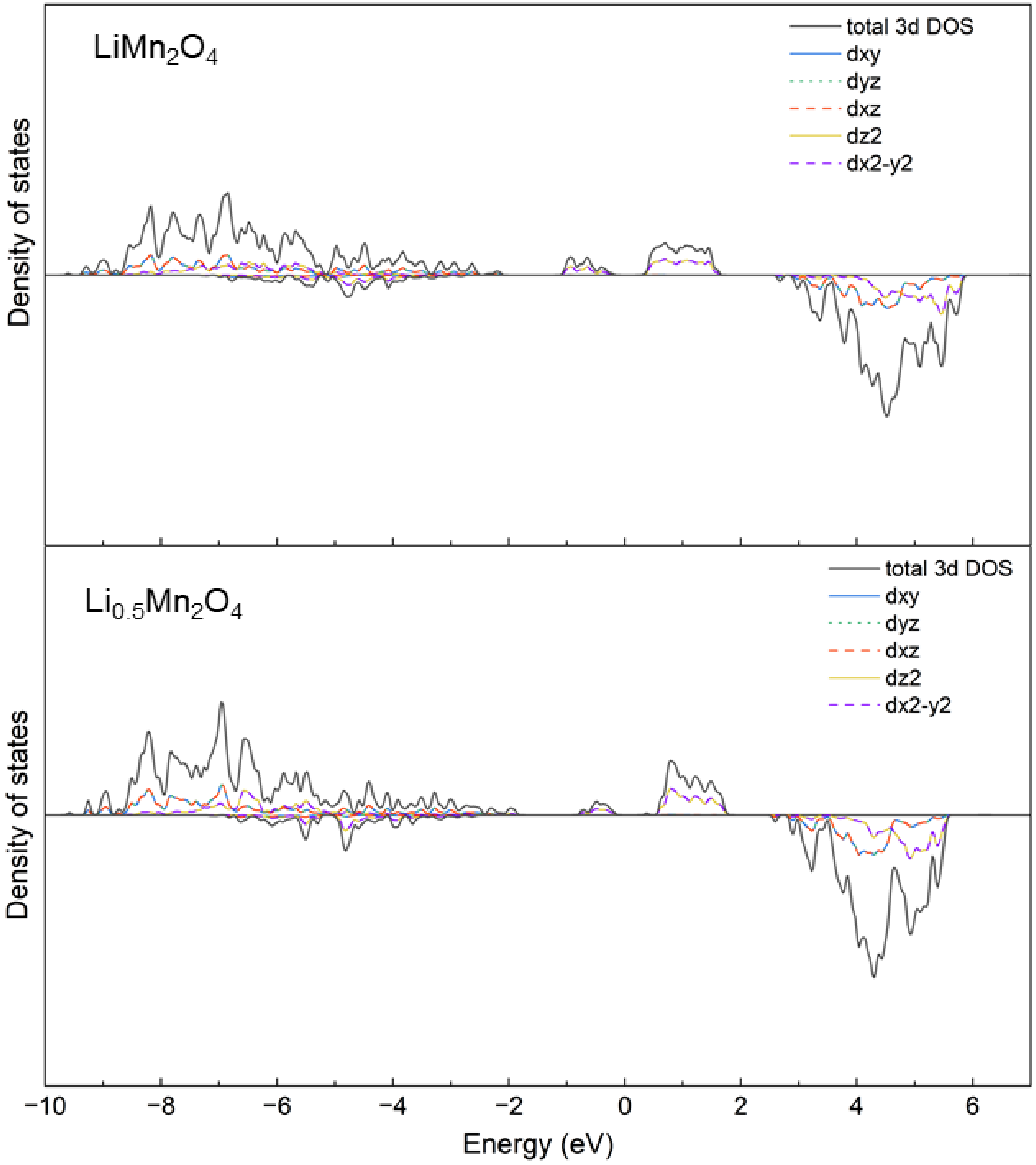

Fig. S8. PDOS of Mn *3d* state of $LiMn_2O_4$ and $Li_{0.5}Mn_2O_4$ using HSE, respectively.

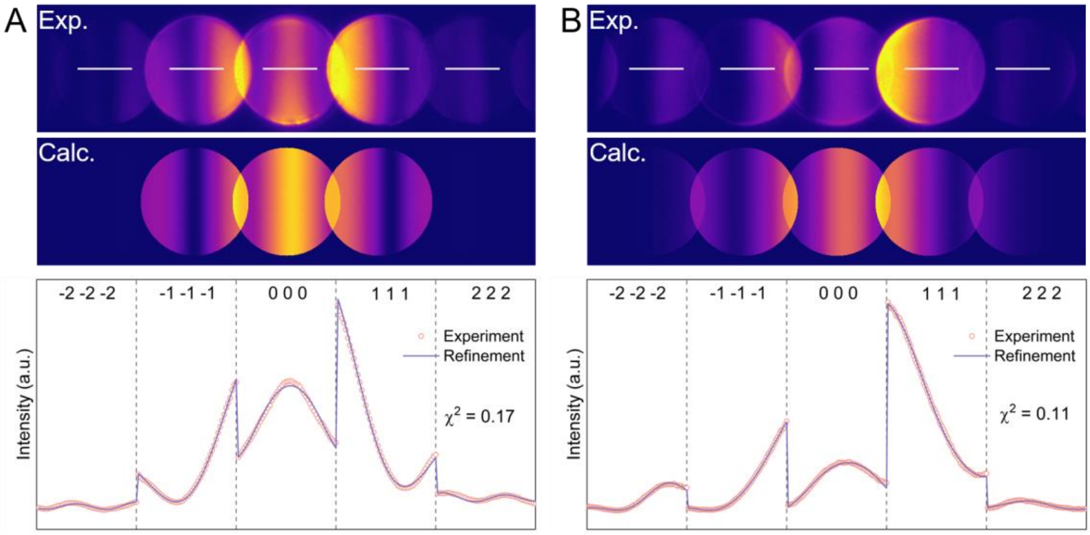

Fig. S9. Measurements of the low-order structure factors from the energy-filtered convergent-beam electron diffraction (111) systematic rows of (A) $LiMn_2O_4$ and (B) $Li_{0.5}Mn_2O_4$. The intensity profiles were extracted from the experimental and refinement results.

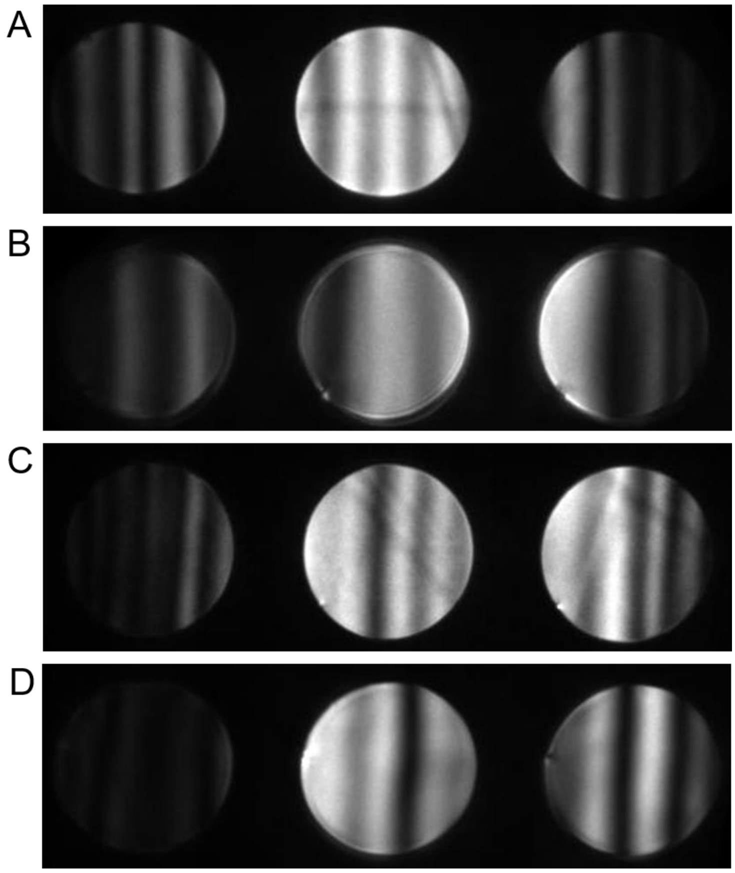

Fig. S10. Comparison of CBED patterns with and without structural changes. (**A-D**) are (004) systematic row CBED patterns of $LiMn_2O_4$. (**A**) is the diffraction pattern without structure change. The messy contrast and crooked rocking curves in (**B**), and the sideling fringes in (**C-D**) indicate the structure changes during beam radiation.

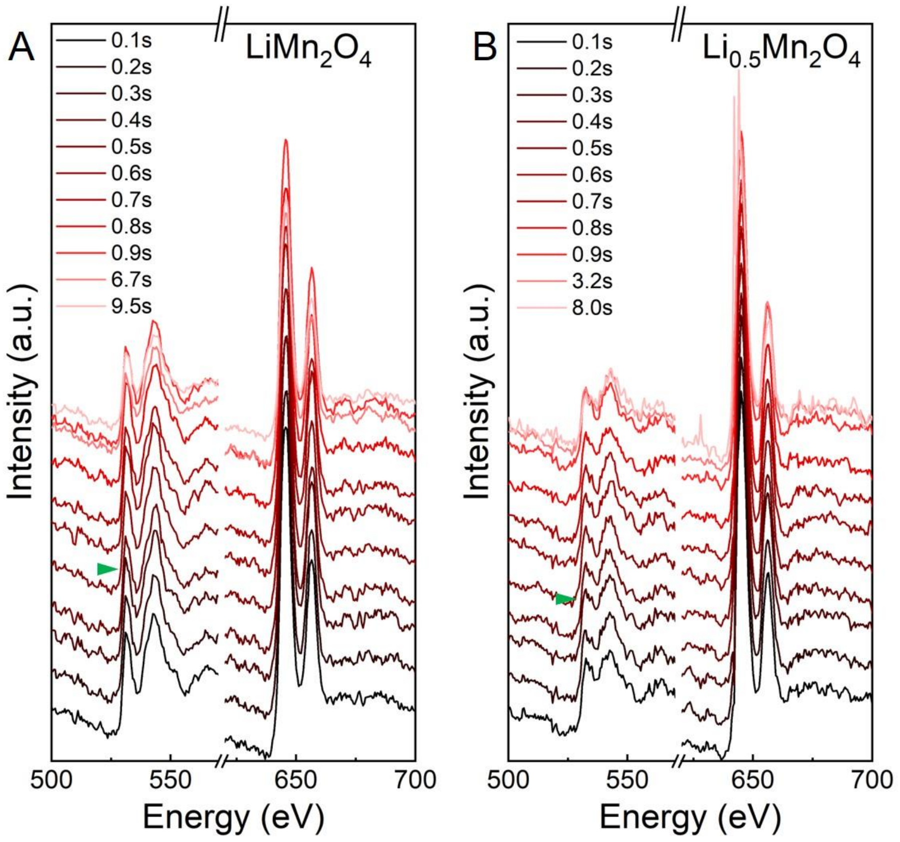

Fig. S11. EELS spectra of the (A) $LiMn_2O_4$ and (B) $Li_{0.5}Mn_2O_4$. Electronic structures changed at 0.3s as indicated by the green triangle.

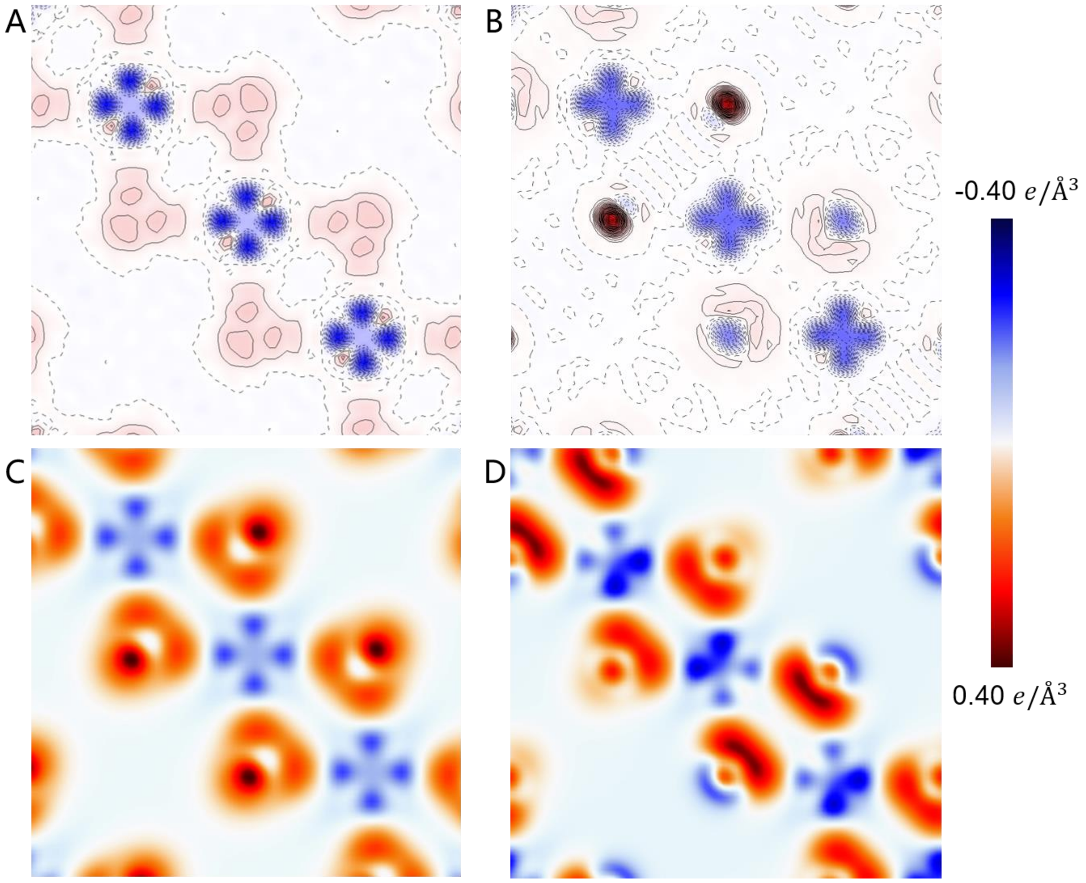

Fig. S12. Experimental static deformation electron density maps of the (001) MnO_4 plane of (A) $LiMn_2O_4$ and (B) $Li_{0.5}Mn_2O_4$. The contour interval is 0.1 *e* □$^{-3}$, with positive and negative contours drawn as solid red and dashed blue lines, respectively. **(C-D)** The corresponding difference electron density map between the calculated and the neutral atoms of $LiMn_2O_4$ and $Li_{0.5}Mn_2O_4$, respectively.

Table S1. Low-order structure factors measurement using CBED for $LiMn_2O_4$.

Reflection	F_e^{CBED}	χ^2	F_X^{Con}	F_X^{DFT}
111	42.129	0.097	195.34	195.66
022	-9.071	0.125	-19.53	-20.62
113	-29.496	3.875	-154.66	-153.49
222	16.001	0.073	124.83	123.28
004	76.822	1.514	419.10	420.38

Table S2. Low-order structure factors measurement using CBED for $Li_{0.5}Mn_2O_4$.

Reflection	F_e^{CBED}	χ^2	F_X^{Con}	F_X^{DFT}
111	42.895	0.090	203.46	201.68
022	-4.868	5.951	-12.93	-13.26
113	-27.198	1.349	-149.06	-148.29
222	17.750	0.108	120.7	124.01
004	76.408	0.983	429.11	426.38

Table S3. Structure factors used in the multipole refinements for $LiMn_2O_4$ and $Li_{0.5}Mn_2O_4$.

h k l	$\|F\|_{Li1}$	$\|F\|_{Li0.5}$
-1 -1 -1	195.19	203.46
0 -2 -2	19.53	12.93
-1 -1 -3	154.66	149.06
-2 -2 -2	124.83	120.7
0 0 -4	419.10	429.11
-1 -3 -3	96.36	101.13
-2 -2 -4	8.61	4.42
-3 -3 -3	86.04	82.05
-1 -1 -5	164.55	160.58
0 -4 -4	345.31	339.52
-1 -3 -5	116.72	120.39
-2 -4 -4	11.33	13.7
0 -2 -6	2.39	2.59
-3 -3 -5	109.71	105.91
-2 -2 -6	124.92	124.7
-4 -4 -4	268.04	272.71
-1 -5 -5	126.49	129.63
-1 -1 -7	73.71	77.52
-2 -4 -6	14.95	10.91
-3 -5 -5	117.55	114.29
-1 -3 -7	72.58	69.62
0 0 -8	247.11	243.36
-3 -3 -7	46.36	50.36
-4 -4 -6	7.35	7.59
0 -6 -6	24.25	19.96
-2 -2 -8	9.25	7.92
-1 -5 -7	84.78	81.98
-5 -5 -5	126.35	123.84
-2 -6 -6	112.08	111.58
0 -4 -8	206.09	209.95
-3 -5 -7	65.33	67.93
-1 -1 -9	99.98	102.46
-2 -4 -8	12.74	12.46
-4 -6 -6	6.64	10.44
-1 -3 -9	96.98	94.49
-4 -4 -8	201.74	198.41
-1 -7 -7	44.5	47.37
-5 -5 -7	72.16	74.29
-3 -3 -9	78.42	80.48
-2 -6 -8	2.81	5.6
0 -2 -10	13.63	10.42
-3 -7 -7	42.3	40.57
-1 -5 -9	105.06	103.07
-6 -6 -6	102.77	102.07
-2 -2 -10	100.12	99.89
-3 -5 -9	84.51	86.31
-4 -6 -8	10.29	12.71
-2 -4 -10	0.88	4.68
-5 -7 -7	60.1	57.85
-1 -1 -11	50.67	49.01
0 -8 -8	173.9	171.16
-1 -7 -9	67.09	68.71
-5 -5 -9	95.6	97.48
-1 -3 -11	38	40.62
-2 -8 -8	19.87	19.8

-4 -4 -10	4.35	11.69
0 -6 -10	14.13	16.47
-6 -6 -8	14.19	12.93
-3 -7 -9	70.47	68.34
-3 -3 -11	37.96	36.69
-2 -6 -10	95.72	95.17
-4 -8 -8	155.05	157.25
0 0 -12	149.1	151.92
-1 -5 -11	47.04	48.96
-7 -7 -7	25.1	28.37
-4 -6 -10	21.82	19.65
-2 -2 -12	3.93	7.18
-5 -7 -9	72.01	70.18
-3 -5 -11	51.21	49.54
0 -4 -12	151.99	150.01
-1 -9 -9	85.27	86.8
-6 -8 -8	16.92	16.57
-2 -4 -12	13.35	13.46
-2 -8 -10	8.83	8.65
-3 -9 -9	81.35	79.87
-1 -7 -11	39.57	38.31
-5 -5 -11	53.97	52.39
-1 -1 -13	85.12	83.77
-6 -6 -10	92.75	92.05
-4 -4 -12	138.14	140.2
-3 -7 -11	25.97	28.69
-7 -7 -9	56.41	57.47
-1 -3 -13	73.23	74.44
-4 -8 -10	7.07	8.39
-2 -6 -12	5.73	8.11
-5 -9 -9	92.72	91.79
-3 -3 -13	75.13	73.5
-8 -8 -8	143.81	141.65
-5 -7 -11	42.01	43.4
-1 -5 -13	79.35	80.68
-4 -6 -12	11.5	11.32
0 -10 -10	29.66	27.79
-6 -8 -10	9.34	12.41
0 -2 -14	3.76	5.5
-1 -9 -11	54.47	52.97
-3 -5 -13	76.8	75.55
-2 -10 -10	90.33	89.61
-2 -2 -14	90.26	89.84
0 -8 -12	128.84	130.45
-3 -9 -11	49.85	50.83
-7 -9 -9	60.83	61.71
-2 -8 -12	23.42	22.87
-4 -10 -10	20.71	22.5
-6 -6 -12	2.08	8.2
-2 -4 -14	9.96	8.81
-1 -7 -13	67.47	65.99
-7 -7 -11	25.44	25.34
-5 -5 -13	85.49	84.74
-4 -8 -12	131.61	129.87
-5 -9 -11	48.06	48.91
-3 -7 -13	62.52	63.19
-1 -1 -15	29.86	31.71

-8 -8 -10	12.15	13.94
-4 -4 -14	2.22	7.76
0 -6 -14	23.2	21.64
-1 -3 -15	32.8	32.17
-6 -10 -10	88.89	88.04
-2 -6 -14	89.31	88.69
-1 -11 -11	31.1	32.8
-5 -7 -13	60.22	60.94
-9 -9 -9	83.06	84.46
-3 -3 -15	24.92	27.19
-6 -8 -12	20.33	20.32
-2 -10 -12	0.02	7.51
-4 -6 -14	15.77	17.42
-3 -11 -11	27.73	27.55
-1 -9 -13	77.95	77.31
-7 -9 -11	52.74	51.28
-1 -5 -15	36.85	35.95
0 0 -16	115.64	114.57
-3 -9 -13	66.18	67.1
-3 -5 -15	33.89	35.2
-4 -10 -12	8.37	10.41
-2 -8 -14	2.54	6.73
-8 -10 -10	21.24	20.74
-2 -2 -16	2.87	8.62
-5 -11 -11	42.99	41.88
-7 -7 -13	62.83	61.3
-6 -6 -14	88.18	87.4
-8 -8 -12	115.69	116.66
0 -4 -16	108.24	109.69
-5 -9 -13	76.86	78.13
-1 -7 -15	28.95	30.63
-5 -5 -15	33.12	34.14
-4 -8 -14	3.83	9.84
-2 -4 -16	13.32	13.01
-6 -10 -12	10.42	11.92
-3 -7 -15	27.01	27.01
-9 -9 -11	48.98	47.8
0 -12 -12	116.03	114.62
-4 -4 -16	111.09	109.97
-1 -11 -13	50.96	51.38
-7 -11 -11	19.01	22.02
-1 -1 -17	68.84	69.63
-2 -12 -12	29.17	28.53
0 -10 -14	26.59	27.79
-6 -8 -14	16.85	16.7
-2 -6 -16	1.6	8.71
-3 -11 -13	56.44	55.07
-7 -9 -13	58.06	57.14
-5 -7 -15	38.81	37.95
-1 -3 -17	69.24	68.26
-2 -10 -14	86.87	86.04
-10 -10 -10	86.22	85.32
-4 -12 -12	108.92	109.78
-1 -9 -15	36.43	37.13
-3 -3 -17	65.46	65.84
-8 -10 -12	14.9	14.9
-4 -6 -16	11.6	11.8

-4 -10 -14	28.96	28.1
-5 -11 -13	50.19	49.05
-3 -9 -15	42.29	41.24
-1 -5 -17	70.64	70.14
0 -8 -16	106.48	105.35
-7 -7 -15	20.49	23.16
-9 -11 -11	46.41	46.77
-3 -5 -17	62.92	63.58
-6 -12 -12	25.38	24.76
-2 -8 -16	24.01	23.48
-8 -8 -14	6.72	8.83
-6 -6 -16	4.1	9.4
0 -2 -18	8.85	7.85
-5 -9 -15	36.85	35.86
-9 -9 -13	77.24	77.21
-6 -10 -14	85.51	84.63
-2 -2 -18	89.38	88.69
-4 -8 -16	100.46	101.32
-1 -13 -13	65.07	66.13
-7 -11 -13	53.35	53.44
-1 -7 -17	59.6	59.87
-5 -5 -17	68.19	69.23
-2 -12 -14	5.2	8.73
-10 -10 -12	9.79	13.2
-2 -4 -18	4.39	6.13
-3 -13 -13	60.39	60.02
-1 -11 -15	32.87	32.54
-3 -7 -17	63.99	62.75
-8 -12 -12	105.88	104.58
-3 -11 -15	24.01	26.08
-7 -9 -15	41.72	42.11
-4 -12 -14	4.64	7.02
-6 -8 -16	20.89	20.37
-4 -4 -18	0.52	10.13
-2 -10 -16	2.92	9.22
-8 -10 -14	19.32	21.07
0 -6 -18	17.23	18.22
-5 -13 -13	70.99	71.04
-5 -7 -17	57.5	56.65
-11 -11 -11	19.72	21.1
-1 -1 -19	26.35	26.17
-2 -6 -18	87.55	86.75
-5 -11 -15	37	37.67
-1 -9 -17	62.32	63.28
-9 -11 -13	42.22	42.5
-1 -3 -19	24.77	26.29
-4 -10 -16	8.5	8.7
-6 -12 -14	7.48	11.32
-4 -6 -18	19.43	18.89
-3 -9 -17	58.9	58.53
-3 -3 -19	25.95	26.19
-8 -8 -16	98.42	97.31
-7 -13 -13	47.65	48.2
-9 -9 -15	32.05	32.31
-7 -7 -17	59.64	59.53
-1 -5 -19	24.59	25.61
-10 -12 -12	18.62	18.73

0 -14 -14	34.24	33.61
-6 -10 -16	1.5	9.27
-2 -8 -18	6.64	8.08
-1 -13 -15	41.5	40.44
-7 -11 -15	22.02	23.13
-5 -9 -17	66.75	66.82
-3 -5 -19	29.81	29.4
-2 -14 -14	82.9	81.98
-10 -10 -14	82.44	81.47
-6 -6 -18	85.6	84.73
0 -12 -16	92.71	93.23
0 0 -20	82.6	83.54

Table S4. Electron beam current and dose of the two different experimental conditions.

	111/222	others
Convergent Semi-angle (mrad)	3.10	4.30
Electron beam current (nA)	0.11	0.12
Spot diameter (*nm*)	1.0	0.7
Dose ($e/s \cdot Å^2$)	8.75×10^6	1.94×10^7

Table S5. Results from multipole refinement of $LiMn_2O_4$.

Parameters	$LiMn_2O_4$
$(sin\theta/\lambda)_{max}$ ($Å^{-1}$)	1.21
wR(F^2) (%)	0.47
N_{refl}	238
P_v(Mn)	4.739(45)
P_v(O)	6.380(22)
P_{20}, P_{40}, P_{43-}(Mn)	-0.0029(34), 0.1924(50), -0.1554(71)
P_{10}, P_{20}, P_{30}, P_{33-}, P_{40}, P_{43}(O)	-0.0425(218), 0.0000(94), -0.0825(190), 0.1007(225), -0.0385(135), 0.0605(187)
κ, κ'(Mn)	0.9977 (95), 2.2956(260)
κ, κ'(O)	0.9873(30), 0.6434(450)

Table S6. Results from multipole refinement of $Li_{0.5}Mn_2O_4$.

Parameters	$Li_{0.5}Mn_2O_4$
$(sin\theta/\lambda)_{max}$ (Å^{-1})	1.21
wR(F^2) (%)	0.58
N_{refl}	260
P_v (Mn)	4.5940(1035)
P_v (O1)	6.5021(1032)
P_v (O2)	6.1539(1038)
P_{10}, P_{20}, P_{30}, P_{33-}, P_{40}, P_{43-} (Mn)	0.0015(31), -0.0142(36), -0.2112(762), -0.1655(625), 0.1795(93), -0.1317(103)
P_{10}, P_{20}, P_{30}, P_{33-}, P_{40}, P_{43-} (O1)	0.0627(347), 0.0076(98), -0.0002(186), 0.0478(160), -0.0191(180), -0.0211(104)
P_{10}, P_{20}, P_{30}, P_{33-}, P_{40}, P_{43-} (O2)	0.1020(16), -0.0194(85), 0.0210(173), 0.0456(141), 0.0292(137), 0.0371(108)
κ, κ'(Mn)	0.9575(130), 2.2575(466)
κ, κ'(O1)	0.9552(74), 1.7335(337)
κ, κ'(O2)	1.0115(89), 2.2694(4927)

Table S7. Mn-O bond lengths in blue and purple MnO_6 octahedra for $NaLi_{0.2}Mn_{0.8}O_2$ from DFT calculation.

Blue MnO_6 octahedron (Å)	Purple MnO_6 octahedron (Å)
1.95	1.95
1.95	1.96
2.00	1.96
2.02	1.96
2.24	2.00
2.24	2.00

Table S8. The TM–O bond length and TMO_6 distortion for spinel $Li_{1-x}TM_2O_4$.

Elements	Bond length (Å)		
M	(6, 0)-LiM_2O_4	(3, 3)-$Li_{0.5}M_2O_4$	(2, 4)-$Li_{0.5}M_2O_4$
Ti	2.01723	2.02154 1.98252	1.97755 2.01477
V	2.00191	2.00684 1.9734	1.8771 2.03079
Cr	1.99003	1.98315 1.95596	1.89973 1.99302
Mn	1.97193	1.97255 1.94663	1.94025 2.00767
Fe	2.00429	1.99858 1.97508	1.97523 2.06448
Co	1.89669	1.89826 1.87398	1.86749 1.89738
Ni	1.91863	1.89665 1.88297	1.91153 1.94204
Cu	1.97752	1.96881 1.95149	1.9497 1.9784
Zn	2.03997	2.0314 2.02943	2.01072 2.06257

Table S9. The TM–O bond length and TMO_6 distortion for perovskite $La(Li_xTM_{1-x})O_3$.

Elements	Bond length (Å)	
M	(6, 0)-$LaMO_3$	(2, 4)-$La(Li_{0.167}M_{0.833})O_3$
Ti	1.98074	1.93464 1.97972
V	1.93943	1.93623 1.89296
Cr	1.96204	1.88016 1.95382
Mn	1.97463	1.89295 1.92266
Fe	1.96748	1.87987 1.9811
Co	1.90663	1.84571 1.93554
Ni	1.92505	1.84344 1.87206
Cu	1.94156	1.86287 2.01291
Zn	1.96224	1.92922 1.97527

Table S10. Structures containing the (6, 0) or (3, 3) configurations of MX_6 octahedra, where is no JT distortion.

Compound	space group	point group	crystal system	Distortion index (%) *(D)*	Reference number
$SrTiO_3$	$Pm\bar{3}m$	O_h	cubic	0	15
$CaTiO_3$	$Pm\bar{3}m$	O_h	cubic	0	16
$LaTiO_3$	$Pm\bar{3}m$	O_h	cubic	0	17
$LaVO_3$	$Pm\bar{3}m$	O_h	cubic	0	18
CdV_2O_4	$Fd\bar{3}m$	O_h	cubic	0	19
KVF_3	Pm-3m	O_h	cubic	0	20
$FeCr_2S_4$	$Fd\bar{3}m$	O_h	cubic	0	21
$LaMnO_3$	$Pm\bar{3}m$	O_h	cubic	0	22
$LiMn_2O_4$	$Fd\bar{3}m$	O_h	cubic	0	23
$SrCoO_3$	$Pm\bar{3}m$	O_h	cubic	0	24
$LaCoO_3$	$Pm\bar{3}m$	O_h	cubic	0	25
$LaNiO_3$	$R\bar{3}c$	D_{3d}	trigonal	0	26
$KZnF_3$	$Pm\bar{3}m$	O_h	cubic	0	27

Distortion Index: $D = \frac{1}{6}\sum_{i=1}^{6}\frac{|l_i - l_{av}|}{l_{av}} \times 100\%$, which is based on the bond length[28]. l_{av} is the average bond length and l_i is the bond length from the *i*th ligand atom to the center transition-metal atoms in the MX_6 octahedron.

Table S11. Structures containing the (2, 4) configuration of MX_6 octahedra, where there is JT distortion.

Compound	space group	point group	crystal system	Distortion index (%)	Reference number
$LaTiO_3$	$Pnma$	D_{2h}	orthorhombic	0.346	29
$YTiO_3$	$Pnma$	D_{2h}	orthorhombic	1.321	30
CdV_2O_4	$I4/amd$	D_{4h}	tetragonal	3.013	31
$LaVO_3$	$I4/mcm$	D_{4h}	tetragonal	1.338	32
YVO_3	$P21/c$	C_{2h}	monoclinic	5.121	33
$KCrF_3$	$I4/mcm$	D_{4h}	tetragonal	6.327	34
$NdFe_{0.2}Mn_{0.8}O_3$	$Pnma$	D_{2h}	orthorhombic	4.616	35
$La_{0.65}Ca_{0.35}MnO_3$	$Pnma$	D_{2h}	orthorhombic	0.549	36
$NdFe_{0.5}Mn_{0.5}O_3$	$Pnma$	D_{2h}	orthorhombic	2.151	35
Cs_2MnF_4	$I4/mmm$	D_{4h}	tetragonal	1.665	37
$LaMnO_3$	$Pnma$	D_{2h}	orthorhombic	3.054	38
$CaMnO_3$	$Pnma$	D_{2h}	orthorhombic	0.386	39
$YMnO_3$	$Pnma$	D_{2h}	orthorhombic	6.638	40
$La_{0.9}Sr_{0.1}MnO_3$	$Pnma$	D_{2h}	orthorhombic	1.310	41
$BiFeO_3/LaFeO_3$	$Pnma$	D_{2h}	orthorhombic	0.405	42
Rb_2CuCl_4	$Cmce$	D_{2h}	orthorhombic	8.085	43
$KCuF_3$	$P4/mmm$	D_{4h}	tetragonal	2.299	44
La_2CuMnO_6	$Pnma$	D_{2h}	orthorhombic	1.833	45
La_2NiO_4	$I4/mmm$	D_{4h}	tetragonal	6.561	46

Distortion Index: $D = \frac{1}{6}\sum_{i=1}^{6}\frac{|l_i - l_{av}|}{l_{av}} \times 100\%$, which is based on the bond length[28]. l_{av} is the average bond length and l_i is the bond length from the ith ligand atom to the center transition-metal atoms in the MX_6 octahedron.

Reference

1. Ma C, Wu L, Yin W-G, Yang H, Shi H, Wang Z, *et al.* Strong Coupling of the Iron-Quadrupole and Anion-Dipole Polarizations in Ba(Fe1-xCox)(2)As-2. *Physical Review Letters* 2014, **112**(7).
2. Blaha; P, Schwarz; K, Madsen; GKH, Kvasnicka; D, Luitz; J, Laskowski; R, *et al.* *WIEN2K: An Augmented Plane Wave Plus Local Orbitals Program for Calculating Crystal Properties*, 2021.
3. Blaha P, Schwarz K, Sorantin P, Trickey SB. Full-potential, linearized augmented plane wave programs for crystalline systems. *Computer Physics Communications* 1990, **59**(2)**:** 399-415.
4. Zuo JM, Weickenmeier AL. ON THE BEAM SELECTION AND CONVERGENCE IN THE BLOCH-WAVE METHOD. *Ultramicroscopy* 1995, **57**(4)**:** 375-383.
5. Coppens P. *X-ray charge densities and chemical bonding*. Oxford University Press: International Union of Crystallography, 1997.
6. Koritsanszky TS, Coppens P. Chemical Applications of X-ray Charge-Density Analysis. *Chemical Reviews* 2001, **101**(6)**:** 1583-1628.
7. Kurki‐Suonio K. IV. Symmetry and its Implications. *Israel Journal of Chemistry* 1977, **16**(2‐3)**:** 115-123.
8. Václav P, Michal D, Lukáš P. Crystallographic Computing System JANA2006: General features. *Zeitschrift für Kristallographie - Crystalline Materials* 2014, **229**(5)**:** 345-352.
9. Hansen NK, Coppens P. Testing aspherical atom refinements on small-molecule data sets. *Acta Crystallographica Section A* 1978, **34**(6)**:** 909-921.
10. Stevens ED, Coppens P. Refinement of metal d-orbital occupancies from X-ray diffraction data. *Acta Crystallographica Section A* 1979, **35**(4)**:** 536-539.

11. Spence JCH, Zuo JM. *Electron Microdiffraction*. Springer Science+Business Media: New York, 1992.

12. Blöchl PE. Projector augmented-wave method. *Physical Review B* 1994, **50**(24)**:** 17953-17979.

13. Kresse G, Furthmüller J. Efficient iterative schemes for ab initio total-energy calculations using a plane-wave basis set. *Physical Review B* 1996, **54**(16)**:** 11169-11186.

14. Kresse G, Furthmüller J. Efficiency of ab-initio total energy calculations for metals and semiconductors using a plane-wave basis set. *Computational Materials Science* 1996, **6**(1)**:** 15-50.

15. Drożdż E, Łącz A, Koleżyński A, Mikuła A, Mars K. Experimental and theoretical studies of structural and electrical properties of highly porous Sr1-xYxTiO3. *Solid State Ionics* 2017, **302:** 173-179.

16. Yashima M, Ali R. Structural phase transition and octahedral tilting in the calcium titanate perovskite CaTiO3. *Solid State Ionics* 2009, **180**(2)**:** 120-126.

17. Wang FB, Li J, Wang P, Zhu XH, Zhang MJ, Peng ZH, *et al.* Effect of oxygen content on the transport properties of LaTiO3+β/2 thin films. *Journal of Physics: Condensed Matter* 2006, **18**(26)**:** 5835.

18. Mahajan AV, Johnston DC, Torgeson DR, Borsa F. Structural, electronic, and magnetic properties of LaxSr1-xVO3 (0.1≤x≤1.0). *Physical Review B* 1992, **46**(17)**:** 10973-10985.

19. Kiswandhi A, Ma J, Brooks JS, Zhou HD. Effects of inter-vanadium distance and A-site magnetism in AV2O4 (A=Cd, Mg, Zn) spinels near the itinerant electron limit. *Physical Review B* 2014, **90**(15)**:** 155132.

20. Williamson RF, Boo WOJ. Lower valence fluorides of vanadium. 1. Synthesis and characterization of sodium trifluorovanadate, potassium trifluorovanadate, and rubidium trifluorovanadate. *Inorganic Chemistry* 1977, **16**(3)**:** 646-648.

21. Amiel Y, Rozenberg GK, Nissim N, Milner A, Pasternak MP, Hanfland M, *et al.* Intricate relationship between pressure-induced electronic and structural transformations in FeCr2S4. *Physical Review B* 2011, **84**(22)**:** 224114.

22. Córdoba JM, Ponce M, Sayagués MJ. Structure evolution in the LaMn1−xFexO3+δ system by Rietveld analysis. *Solid State Ionics* 2017, **303:** 132-137.

23. Hunter JC. Preparation of a new crystal form of manganese dioxide: λ-MnO2. *Journal of Solid State Chemistry* 1981, **39**(2)**:** 142-147.

24. Long Y, Kaneko Y, Ishiwata S, Taguchi Y, Tokura Y. Synthesis of cubic SrCoO3 single crystal and its anisotropic magnetic and transport properties. *Journal of Physics: Condensed Matter* 2011, **23**(24)**:** 245601.

25. Haas O, Struis RPWJ, McBreen JM. Synchrotron X-ray absorption of LaCoO3 perovskite. *Journal of Solid State Chemistry* 2004, **177**(3)**:** 1000-1010.

26. Zhang J, Zheng H, Ren Y, Mitchell JF. High-Pressure Floating-Zone Growth of Perovskite Nickelate LaNiO3 Single Crystals. *Crystal Growth & Design* 2017, **17**(5)**:** 2730-2735.

27. Knight KS, Bull CL, McIntyre P. Low temperature, high pressure thermo-physical and crystallographic properties of KZnF3 perovskite. *Materials Chemistry and Physics* 2017, **199:** 393-407.

28. Baur WH. The geometry of polyhedral distortions. Predictive relationships for the phosphate group. 1974, **30**(5)**:** 1195-1215.

29. Hemberger J, von Nidda HAK, Fritsch V, Deisenhofer J, Lobina S, Rudolf T, *et al.* Evidence for Jahn-Teller Distortions at the Antiferromagnetic Transition in LaTiO3. *Physical Review Letters* 2003, **91**(6)**:** 066403.

30. Kibalin IA, Yan Z, Voufack AB, Gueddida S, Gillon B, Gukasov A, *et al.* Spin density in YTiO3: I. Joint refinement of polarized neutron diffraction and magnetic x-ray diffraction data leading to insights into orbital ordering. *Physical Review B* 2017, **96**(5)**:** 054426.

31. Masashige O, Junichi H. A distortion of pseudotetramers coupled with the Jahn–Teller effect in the geometrically frustrated spinel system CdV2O4. *Journal of Physics: Condensed Matter* 2003, **15**(3)**:** L95.

32. Nakamura T, Petzow G, Gauckler LJ. Stability of the perovskite phase LaBO3 (B = V, Cr, Mn, Fe, Co, Ni) in reducing atmosphere I. Experimental results. *Materials Research Bulletin* 1979, **14**(5)**:** 649-659.

33. Blake GR, Palstra TTM, Ren Y, Nugroho AA, Menovsky AA. Transition between Orbital Orderings in YVO3. *Physical Review Letters* 2001, **87**(24)**:** 245501.

34. Margadonna S, Karotsis G. Cooperative Jahn-Teller Distortion, Phase Transitions, and Weak Ferromagnetism in the KCrF3 Perovskite. *Journal of the American Chemical Society* 2006, **128**(51)**:** 16436-16437.

35. Chakraborty T, Yadav R, Elizabeth S, Bhat HL. Evolution of Jahn–Teller distortion, transport and dielectric properties with doping in perovskite NdFe1−xMnxO3 ($0 \le x \le 1$) compounds. *Physical Chemistry Chemical Physics* 2016, **18**(7)**:** 5316-5323.

36. Dai P, Zhang J, Mook HA, Liou SH, Dowben PA, Plummer EW. Experimental evidence for the dynamic Jahn-Teller effect in La0.65Ca0.35MnO3. *Physical Review B* 1996, **54**(6)**:** R3694-R3697.

37. Iwasa K, Nishi M, Ikeda H, Suzuki J-i. Pressure-Induced Long-Period Magnetic Structure in Layered Antiferromagnets Rb2MnF4 and Cs2MnF4. *Journal of the Physical Society of Japan* 1994, **63**(5)**:** 1900-1907.

38. Thygesen PMM, Young CA, Beake EOR, Romero FD, Connor LD, Proffen TE, *et al.* Local structure study of the orbital order/disorder transition in LaMnO3. *Physical Review B* 2017, **95**(17)**:** 174107.

39. Abrashev MV, Bäckström J, Börjesson L, Popov VN, Chakalov RA, Kolev N, *et al.* Raman spectroscopy of CaMnO3: Mode assignment and relationship between Raman line intensities and structural distortions. *Physical Review B* 2002, **65**(18)**:** 184301.

40. Okuyama D, Ishiwata S, Takahashi Y, Yamauchi K, Picozzi S, Sugimoto K, *et al.* Magnetically driven ferroelectric atomic displacements in orthorhombic YMnO3. *Physical Review B* 2011, **84**(5)**:** 054440.

41. Louca D, Egami T, Brosha EL, Röder H, Bishop AR. Local Jahn-Teller distortion in La1-xSrxMnO3 observed by pulsed neutron diffraction. *Physical Review B* 1997, **56**(14)**:** R8475-R8478.

42. Sun W, Wang W, Chen D, Cheng Z, Jia T, Wang Y. Giant Magnetoelectric Coupling and Two-Dimensional Electron Gas Regulated by Polarization in BiFeO3/LaFeO3 Heterostructures. *The Journal of Physical Chemistry C* 2019, **123**(26)**:** 16393-16399.

43. Aguado F, Rodríguez F, Valiente R, Itiè J-P, Hanfland M. Pressure effects on Jahn-Teller distortion in perovskites: The roles of local and bulk compressibilities. *Physical Review B* 2012, **85**(10)**:** 100101.

44. Manivannan V, Parhi P, Kramer JW. Metathesis synthesis and characterization of complex metal fluoride, KMF3 (M = Mg, Zn, Mn, Ni, Cu and Co) using mechanochemical activation. *Bulletin of Materials Science* 2008, **31**(7)**:** 987-993.

45. Guo L, Bai Y, Huang C, Ma W. Revisiting La2MMnO6 (M = Co, Ni, Cu, Zn) perovskites in view of 3d-electron configuration. *Journal of Applied Physics* 2018, **124**(6)**:** 065103.

46. Skinner SJ. Characterisation of La2NiO4+δ using in-situ high temperature neutron powder diffraction. *Solid State Sciences* 2003, **5**(3)**:** 419-426.
